# Examining the Effects of Mindful Eating Training on Adherence to a Carbohydrate-Restricted Diet in Patients With Type 2 Diabetes (the DELISH Study): Protocol for a Randomized Controlled Trial

**DOI:** 10.2196/11002

**Published:** 2019-02-20

**Authors:** Ashley E Mason, Laura Saslow, Patricia J Moran, Sarah Kim, Priyanka K Wali, Hiba Abousleiman, Alison Hartman, Robert Richler, Samantha Schleicher, Wendy Hartogensis, Elissa S Epel, Frederick Hecht

**Affiliations:** 1 UCSF Osher Center for Integrative Medicine Department of Medicine University of California San Francisco San Francisco, CA United States; 2 Center for Health and Community Department of Psychiatry University of California San Francisco San Francisco, CA United States; 3 School of Nursing Department of Health Behavior and Biological Sciences University of Michigan Ann Arbor, MI United States; 4 Division of Endocrinology, Diabetes and Metabolism Department of Medicine San Francisco General Hospital San Francisco, CA United States; 5 School of Medicine University of Maryland Baltimore, MD United States

**Keywords:** treatment adherence and compliance, mind-body therapies, diabetes mellitus, diet, ketogenic, mindfulness

## Abstract

**Background:**

Diet patterns have a profound influence on glycemic control for individuals with type 2 diabetes mellitus (T2DM), and craving-related eating is an important obstacle to dietary adherence. A growing body of research suggests that carbohydrate-restricted (CR) diets can improve glycemic control and reduce medication dependence in T2DM. However, limited data speak to the effects of long-term adherence to CR diets. Mindful eating training has been shown to reduce craving-related eating in overweight populations but has yet to be examined as a behavioral support for dietary adherence in T2DM. This trial examines behavioral mechanisms, particularly craving-related eating, through which mindful eating training might improve adherence to CR dietary recommendations in T2DM. This will clarify the importance of focusing on craving-related eating in the optimization of dietary adherence interventions.

**Objective:**

The aim of this trial is to determine whether providing training in mindful eating increases adherence to a CR dietary recommendation in T2DM.

**Methods:**

We are randomizing 60 participants to receive a CR diet with or without mindful eating training (12-week group intervention) and are following participants for 12 weeks after intervention completion. We hypothesize that participants who receive mindful eating training (relative to those who do not) will demonstrate greater adherence to the CR diet.

**Results:**

Our primary outcome is change in craving-related eating, as assessed using an ecological momentary assessment mobile phone–based platform. Secondary behavioral pathway outcomes include changes in stress-related eating, impulsivity, glycemic control, weight change, dietary adherence, and resumption of dietary adherence after dietary nonadherence.

**Conclusions:**

This theory-driven trial will shed light on the impact of mindfulness training on mechanisms that may impact dietary adherence in T2DM.

**Trial Registration:**

ClinicalTrials.gov NCT03207711; https://clinicaltrials.gov/ct2/show/NCT03207711 (Archived by WebCite at http://www.webcitation.org/73pXscwaU)

## Introduction

### Background

Type 2 diabetes mellitus (T2DM) is the costliest chronic disease in the United States, afflicting 30.3 million people in the United States (9.4% of the population) and nearly 382 million people worldwide [1-4]. Lowering glycosylated hemoglobin (HbA_1c_), a central measure of glycemic control in T2DM, reduces the risk of complications such as nephropathy and retinopathy [5]. Although lifestyle modification, particularly in the spheres of nutrition and exercise, is a key component of improving glycemic control in T2DM, achieving long-term adherence to lifestyle changes remains a challenge [6].

The American Diabetes Association currently includes carbohydrate-restricted (CR) diets as one of several appropriate diet patterns for people with T2DM [7]. Several trials, reviews, and meta-analyses suggest that lowering carbohydrate intake can improve glucose control, insulin resistance, and body weight [8-14]. In this trial, we will administer a CR diet that restricts carbohydrates to approximately 10% of caloric intake (about 50 g/day) so as to induce *nutritional ketosis*, a low level of ketone production that serves as a biomarker for assessing dietary adherence to carbohydrate restriction.

Food cravings, defined as intense urges or desires to eat specific types of foods [15], as well as emotional and mindless eating, pose challenges to adherence to diet recommendations, especially for people with T2DM [16,17]. Seeing desirable food can induce food cravings [18-20]; hence, it is unsurprising that food cravings are the most commonly cited reason for dietary nonadherence and that reductions in food cravings are associated with long-term weight loss [21-23]. The modern food environment is replete with food cues and opportunities to eat, which can amplify the impact of food cravings on dietary adherence. Effectively treating T2DM and prediabetes, which together affect more than one-third of Americans, requires long-term dietary adherence [24,25].

Mindful eating practices may increase dietary adherence by reducing craving-related eating [26-28], which poses challenges to individuals with T2DM [16,29-31]. Training in mindfulness, which can be defined as “paying attention on purpose, in the present moment, and nonjudgmentally, to the unfolding of experience moment to moment [32]” may equip individuals with skills to recognize and observe their experiences of food cravings without acting on them (ie, eating in response to them). Mindfulness training focuses on reducing self-judgment (*nonjudgment*), which may increase resilience (resumption of dietary adherence) after temporary lapses in dietary adherence [33]. In this trial, we randomize half of participants to receive training in mindful eating to ascertain whether and how such training may impact behavioral pathways that shape dietary adherence in T2DM.

### Objectives

The primary behavioral change we are testing is whether training in mindful eating can reduce craving-related eating as assessed using ecological momentary assessment (EMA). This trial examines behavioral pathways that we hypothesize to be tied to clinical outcomes; it does not center on clinical outcomes themselves such as improvements in glycemic control. We also test whether mindful eating training may contribute to other adaptive behavioral changes, including (1) decreased impulsivity, (2) decreased stress- and emotion-related eating, and (3) improved resilience after dietary nonadherence occurs (resumption of dietary adherence). We will also test whether mindful eating training may improve glycemic control (HbA_1c_, fasting glucose, and insulin resistance) and contribute to weight loss. We also aim to directly assess whether training in mindful eating improves dietary adherence as assessed in blood ketones and 24-hour dietary recall.

## Methods

### Overview

The central goal of this trial is to test whether there is evidence that supports proposed mechanisms through which our behavioral intervention (a particular type of mindful eating training) impacts eating in response to cravings and/or influences one or more of the additional mechanistic pathways we have hypothesized. We designed this trial in response to a National Institute of Health (NIH) grant announcement designed to investigate mechanisms of action that may lead to change in clinical outcomes. This is a randomized controlled, two-group trial that tests group-based 12-week in-person interventions and follows participants for a 12-week postintervention period (24 weeks of trial participation total). We randomly assign participants to 1 of 2 intervention groups; both groups receive training in how to follow a CR diet, but 1 group also receives mindful eating training. We are recruiting 3 waves of approximately 20 participants each (N=60 total). Participants complete 12 weekly in-person sessions followed by 3 monthly in-person maintenance sessions, for a total of 15 sessions over 6 months. Participants complete self-report questionnaires (online and Qualtrics), provide blood specimen assessments (LabCorp [34] location of choice), and respond to mobile phone–based assessments about their current food cravings and emotions using EMA [35] methods, along with other assessments (see Table 1 for the schedule of evaluations). The University of California, San Francisco (UCSF) institutional review board (IRB16-20025) approved of all trial procedures, and all participants provide written informed consent before enrollment.

### Recruitment and Eligibility

We recruit participants from several sources, including within UCSF clinics (eg, diabetes clinic, general internal medicine clinic). We work with the UCSF Clinical and Translational Science Institute to send physical letters to potentially eligible participants in the UCSF system who had previously consented to be contacted about research studies for which they may be eligible. We also recruit participants via flyers posted in the community as well as use social media strategies, such as Facebook, Nextdoor, and Craigslist (see Textbox 1 for trial inclusion and exclusion criteria.

### Enrollment Procedures

Potential participants make initial contact with the study by visiting the study website or telephoning a dedicated study phone number. They review the study website and the full-study consent before electronically signing an online consent to be screened for study eligibility. They then complete an eligibility Web-based screener (Qualtrics). If potentially eligible per the Web-based screener, study staff conduct a phone screening to further assess eligibility. In this call, study staff confirm basic eligibility criteria and review study schedules, procedures, and assessments. If, after this call, potential participants remain eligible and interested, they then complete a short message service (SMS) text message–based assessment (EMA) that involves responding to SMS text messages from their smartphones 3 times per day, on 3 days distributed across a week. Potential participants must respond to at least 7 of 9 messages and must report eating in response to a craving at least 2 times. If potential participants meet these criteria, they then complete an in-person screening and consent visit. This visit includes collecting or administering (1) health history and medication information, (2) information on excluded substance misuses, mental and medical conditions, and medication contraindications, (3) weight and height assessments, (4) computerized cognitive assessments, and (5) a fasting blood draw to assess parameters required for inclusion: HbA_1c_ between 6.5% and 12.0%, thyroid-stimulating hormone in a normal range, and liver and kidney function in a normal range. We conduct enrollment procedures in the 8 weeks before a wave starts. If the EMA or the HbA_1c_ components of enrollment procedures take place more than 4 weeks before the wave start date, we repeat these measures in the week before the wave start date. Participants remain eligible regardless of data collected during repeat measures.

**Table 1:** Schedule of evaluations and intervention phases.

Time (activity or assessment)	Pre-enrollment (screening and consent visit)	Baseline (preintervention assessment and randomization to intervention arm)	Weeks 0-12 (intervention period)	Week 12 (postintervention assessment)	Weeks 12-24 (maintenance period)	Week 24 (postmaintenance assessment)
Informed consent	✓	—	—	—	—	—
Health history	✓	—	—	—	—	—
Demographics and background information	✓	—	—	—	—	—
Inclusion or exclusion criteria assessment	✓	—	—	—	—	—
Craving EMA^a^	✓	—	✓	✓	✓	✓
Laboratory-based chemistry testing	✓	✓	—	✓	—	✓
Online self-report measures	—	✓	—	✓	—	✓
24-hour dietary recall	—	✓	—	✓	—	✓
Computerized cognitive assessment	✓	—	—	✓	—	✓
Anthropometrics^b^	✓	—	—	✓	—	✓
Weekly diet and/or mindful eating classes	—	—	✓	—	—	—
Monthly diet and/or mindful eating follow-up classes	—	—	—	—	✓	—
Ketone testing for dietary adherence^c^	—	—	✓	✓	✓	✓
Home glucose testing for safety^d^	—	—	✓	—	✓	—

^a^EMA: ecological momentary assessment, administered over 1 week at each pre-enrollment, baseline, and weeks 7, 13, 19, and 24.

^b^Height at baseline only.

^c^Ketone testing 3 times per week during intervention period and 2 times per week during maintenance period.

^d^Only for individuals using insulin and sulfonylurea medications.

**Inclusion and exclusion criteria.**

**Inclusion criteria**

- History of type 2 diabetes
- 6.5%≤glycosylated hemoglobin or HbA_1__c_<12.0%
- Age ≥18 years
- Experience food-related cravings most days of the week and/or eat in response to food cravings more than they wish they did
- On screening ecological momentary assessment (EMA), report eating in response to cravings at least twice over the course of 3 days
- Able to engage in light physical activity
- Have a smartphone and respond to at least 7 of 9 short message service text messages delivered over the course of 3 days during pre-enrollment
- English speaking
- Willing to participate in diet and mindfulness interventions

**Exclusion criteria**

- Unable to provide informed consent
- Substance abuse, mental health, or medical condition that will make it difficult to participate in the intervention or may alter key outcomes or require important diet modifications
- Pregnant or planning to get pregnant in the next 6 months, breastfeeding, or <6 months post­partum
- Current use of weight loss medications or supplements
- History of weight loss (bariatric) surgery in the past 18 months or current plans for bariatric surgery
- Currently enrolled in a weight loss program or have unalterable plans to enroll in one of these programs in the next year
- Vegan or vegetarian
- Unwilling to do home blood ketone monitoring

### Randomization

We randomize participants in a 1:1 fashion to either group using random permuted blocks with varying sizes and stratifying by body mass index (BMI; above and below BMI of 25) after completion of all screening and baseline assessment steps to enrollment and before initiation of intervention. We randomize participants to intervention group approximately 1 week before the start of classes to minimize postrandomization attrition because of life circumstances that may impact class attendance (eg, job loss or family issue).

### Masking

We do not mask participants to intervention group. Some of our assessments, specifically those done at LabCorp (blood draws; [34]) and those done via Web-based self-report surveys, are masked to intervention group. LabCorp [34] locations have no knowledge of the research design or intervention group. In this trial, 24-hour dietary recall interviews are conducted by research assistants blinded to intervention group. We do not mask other study coordinators to intervention group as the funding and study team is not large enough to support separate assessment and intervention teams. No intervention group information is included on the data collection forms used at follow-up visits (eg, weight measurement), but some staff who are highly involved in administrative aspects of the study classes may be aware of intervention group. Study physicians are not strictly blinded to intervention group, but effort is made to limit communication of this information to them. For example, they are not provided intervention group information when they make medication reductions or adjustments either at baseline or during the intervention phase. Participants may, however, in the course of their medication management discussions with study physicians, discuss their experience in the trial and could self-reveal their intervention group. A principal investigator might, in the course of responding to a participant with a study concern, learn of his or her intervention group (eg, if a participant has a concern about the mindful eating intervention that is not adequately addressed by the project director or other key study personnel).

### Trial Period

We have defined the trial period as a 3-level variable, including baseline (the period preceding the intervention), 3 months postbaseline (occurring immediately following the end of the intensive intervention period), and 6 months postbaseline (occurring 6 months after baseline and 3 months after the intensive intervention period ends).

### Data Collection

We collect data at several occasions over the course of the 24-week trial period. Table 1 displays a schedule of evaluations.

### Self-Report Measures

We administer self-report measures of mood, eating behavior, and other psychological factors (online, Qualtrics). Table 2 displays self-report measures.

**Table 2:** Self-report and computerized cognitive assessments.

Type and measure name (acronym; number of items or minutes)		Construct assessed	
**Online (Qualtrics) self-report measures**			
	Perceived Stress Scale (PSS; 10 items) [41]		Global perceptions of stress
	Patient Health Questionnaire (PHQ-8; 8 items) [42]		Depression symptoms
	Reward-based Eating Drive (RED-9; 9 items) [43]		Reward-based drive to eat (loss of control over eating, lack of satiety, and preoccupation with food)
	Food Craving Questionnaire-Trait-Reduced (FCQ-T-R; 15 items) [44]		Emotional food craving, preoccupation with food, loss of control over eating, and positive outcome expectancy
	Midlife in the United States (MIDUS) Stress Eating Items (MIDUS; 2 items) [45]		How one’s eating changes in response to a stressful event: eating to feel better and eating more than usual
	Palatable Eating Motives Scale (PEMS; 19 items) [46]		Social, conformity, enhancement, and coping-based motives for eating tasty food
	Stress Eating (1 item) [47]		Eating when under moderate stress
	Weight Efficacy Lifestyle Questionnaire – Short Form (WEL-SF; 8 items) [48]		Ability to adhere to a diet when in challenging situations (eg, socializing and peer pressure)
	Loss of Control over Eating – Brief (LOCES-Brief; 7 items) [49]		Loss of control over eating
	Food Acceptance and Action Questionnaire (FAAQ; 10 items) [50]		Acceptance of one’s motivations to eat
	Modified Differential Emotions Scale (MDES; 20 items) [51]		Positive and negative emotions
	Dutch Eating Behavior Questionnaire, Restrained Eating Subscale (DEBQ-R; 10 items) [52]		Restrained eating behavior
	Five Factor Mindfulness Questionnaire (FFMQ; 24 items) [53]		Tendencies to be mindful in daily life
	Promis-29 (29 items) [54]		Multidimensional quality of life measure
	Self-Compassion Scale Short Form (SCS-SF13; 12 items) [55]		Self-kindness, self-judgment, common humanity, isolation, mindfulness, over-identification
	Body Responsiveness Questionnaire (BRQ; 7 items) [56]		Responsiveness to bodily sensations
**Computerized cognitive measures**			
	Delayed discounting (DD; 5 trials, 1 min) [57]		Valuation of proximal versus delayed monetary rewards
	Go/No-Go (GNG; 12 min) [58]		Sustained attention and response inhibition
	Food Stroop (5 min) [59]		Food preoccupation
	Relative reinforcing efficacy of food (RRE; 4 min) [60]		Relative reinforcement value of tasty food
	Dot probe (4 min) [61]		Selective attentional processing for food versus nonfood stimuli

### Ecological Momentary Assessment

We assess hunger, stress, craving, craving-related eating, and emotions by sending SMS text messages to participants’ personal smartphones at different points during the trial, as shown in Table 1. Each SMS text message includes a link that participants tap on in order to access the survey, which is administered on the Qualtrics platform. Participants receive SMS text messages 3 times over the course of the day, and these times have yielded high response rates in our prior work [26]. Before participants respond to these SMS text messages, they are presented with the following definition of food craving: “A food craving is a desire to eat a particular type of food or drink. You might go out of your way for it because only specific foods will satisfy the craving. This is different than hunger, because a variety of foods can satisfy hunger.” Our primary outcome is participants’ responses to the question regarding craving-related eating. During enrollment, we review the importance of responding to these SMS text message assessments with participants by explaining that it is difficult for us to understand their daily experiences without hearing firsthand from them, in the moment, what they are experiencing. Participants receive EMA at baseline and at weeks 7, 13, 19, and 24 (see Table 3 for EMA questions and administration schedule [35]).

### Laboratory (Clinical) Assessments

We collect several biomarkers of metabolic health and glycemic control (see Table 4 for blood-based biomarker testing conducted at LabCorp [34]).

### Computerized (Cognitive) Assessments

We administer computerized cognitive assessments (Inquisit) that index metrics, including reward-related behavior, behavioral response inhibition, food preoccupation, and attentional processing. Such factors have emerged as meaningful predictors of eating behavior and weight change over time [36-38] (see Table 2 for computerized cognitive assessments).

**Table 3:** Ecological momentary assessment (EMA) questions.

Time	Questions^a^
11:00 am	1. How physically hungry are you right now? (sliding response: “not at all hungry” to “very hungry”) 2. Since you woke up today, have you craved a particular type of food or drink? (2 choices: “Yes” or “No”) 2a. How strong was that craving? (sliding response: “not strong” to “very strong”) 2b. Did you eat or drink anything in response to that craving (2 choices: “yes” or “no”) 2c. What food or drink did you eat? (open-ended response with a blank text box) 2d. Once you began eating in response to this craving, did you feel you could stop? (sliding response: “I could stop” to “I could NOT stop”)
4:30 pm	1. How physically hungry are you right now? (sliding response: “not at all hungry” to “very hungry”) 2. Since you last responded to one of our texts, have you craved a particular type of food or drink? (2 choices: “yes” or “no”) 2a. How strong was that craving? (sliding response: “not strong” to “very strong”) 2b. Did you eat or drink anything in response to that craving? (2 choices: “yes” or “no”) 2c. What food or drink did you eat? (open-ended response with a blank text box) 2d. Once you began eating in response to this craving, did you feel you could stop? (sliding response: “I could stop” to “I could NOT stop”)
9:00 pm	1-2d questions (identical to 4:30 pm) 3. Today, did you have any other cravings for food or drink that you haven’t yet told us about in one of these texts? (2 choices: “yes” or “no”) 3a. How strong was that craving? (sliding response: “not strong” to “very strong”) 3b. Did you eat or drink anything in response to that craving? (2 choices: “yes” or “no”) 3c. What food or drink did you eat? (open-ended response with a blank text box) 3d Once you began eating in response to this craving, did you feel you could stop? (sliding response: “I could stop” to “I could NOT stop”) 4. Over the entire day, how much have you felt happy/pleased/cheerful? (sliding response: “not at all” to “all the time”) 5. Over the entire day, how much have you felt unhappy/sad/frustrated? (sliding response: “not at all” to “all the time”) 6. Over the course of the entire day, what’s the most stressed you’ve felt? (sliding response: “not at all stressed” to “very stressed”)

^a^The prespecified outcome variable is craving-related eating, as assessed in item 2b (11:00 am, 4:30 pm, and 9:00 pm) and item 3b (11:00 pm only).

**Table 4:** Blood-based chemistry testing (LabCorp).

Test^a^	Construct assessed and/or rationale	Consent visit: eligibility screening	Assessment: baseline	Assessments: 3 and 6 months
Glycosylated hemoglobin	Inclusionary test and study variable: level of overall glucose control	✓	✓	✓
C-peptide	Inclusionary test^b^: confirms type 2 diabetes among individuals using exogenous insulin	✓	—	—
Thyroid stimulating hormone	Exclusionary test: rules out an untreated thyroid disorder	✓	—	—
Comprehensive metabolic panel	Exclusionary test: rules out liver and/or kidney dysfunction; study variable: liver enzymes	✓	✓	✓
Glucose, plasma	Study variable: allows for calculation of insulin resistance and fasting glucose	—	✓	✓
Insulin, plasma	Study variable: allows for calculation of insulin resistance and fasting glucose	—	✓	✓
High-sensitivity C-reactive protein	Study variable: biomarker of inflammation	—	✓	✓
NMR LipoProfile	Study variables: triglycerides and detailed cholesterol measures	—	✓	✓

^a^All samples are collected from individuals in a fasting state.

^b^We assess C-peptide only among potential participants reporting insulin use or a history of diabetic ketoacidosis.

### Dietary Adherence

We measure adherence to carbohydrate restriction in 2 ways: (1) presence of blood ketones, which provide a biological measure of substantial restriction of carbohydrate intake and (2) 24-hour dietary recall.

#### Ketones

In week 4, we provide participants with a home-based blood ketone-monitoring device (Precision Xtra System; Abbott Diabetes Care, Alameda, California) and ketone strips, which participants use with the device to measure β-hydroxybutyrate (BOHB) in the blood. This method was recently used in a CR diet intervention for individuals with T2DM [39]. We teach participants how to use the meter in person and ask them to measure their blood ketones before dinner, 2 to 3 times a week, on alternating days. Participants are asked to report ketone results via online survey on a weekly basis. The meters store data, which staff check at every weekly class (starting in week 5) and every monthly class (starting in week 16) to confirm self-reported measurements. Values collected by meters will be used for analysis; however, if their equipment malfunctions (eg, breakage or loss), we will use available self-reported values. For this trial, we define nutritional ketosis as a BOHB level between 0.3 and 3.0 mmol/L. Although the level of carbohydrate restriction needed to achieve ≥0.3 BOHB varies between individuals, in our experience, most individuals need to restrict carbohydrate intake to below 50 g per day of nonfiber carbohydrate to achieve nutritional ketosis.

#### 24-Hour Dietary Recall

Despite limitations of self-report measures in assessing dietary intake, they remain an important tool to assess dietary adherence. The 24-hour dietary recall provides a measure that complements the ketone measure by providing overall diet composition information that cannot be obtained from ketone measures. We use the University of Minnesota’s Nutrition Data System for Research (NDSR) software to perform 24-hour dietary recalls via telephone. This is a widely used dietary analysis program that enables the collection of multiple 24-hour dietary recalls and encompasses multiple foods appropriate for diets of T2DM patients. Although the NIH recommends a single 24-hour assessment at each repeated time point, we collected 2 assessments at each time point (1 on a weekday and 1 on a weekend day) [40]. Recalls are entered into the NDSR software immediately after completion. Dietary recalls are conducted without prior notification to avoid changes in diet on the reporting day.

### Resilience Following Dietary Nonadherence

Our measure will be the time from a ketone measure of <0.3 BOHB mmol/L to higher levels of ≥0.3 BOHB mmol/L, indicating a return to nutritional ketosis after a period of consuming foods that depress ketosis.

### Interventions

#### Carbohydrate-Restricted (CR) Diet Classes

We teach participants an *ad libitum* CR eating plan, similar to that in our previously published work, which was developed with clinicians, dieticians, and researchers with expertise in this area [8,62]. For this trial’s CR diet, we instruct participants to reduce their carbohydrate intake to between 20 and 35 nonfiber grams of carbohydrates per day (with the goal of remaining under 50 nonfiber grams per day), to eat an adequate amount of protein (as described by the Institute of Medicine [63]), and to eat fat to achieve satiety. This recommendation fits macronutrient profiles that have been demonstrated effective in improving glycemic control among individuals with T2DM in meta-analyses of RCTs [13,64,65]. We advocate a gradual transition toward this CR diet by instructing participants to change different types of meals each week: They change their breakfasts and snacks in the first week, their lunches in the second week, and their dinners in the third week. After approximately 4 weeks, participants are fully implementing CR dietary recommendations. The specific content of participants’ diets varies but generally includes green leafy and other nonstarchy vegetables, nuts, seeds, fats (except trans fats), fish, poultry, meats, eggs, cheese, avocados, and low-carbohydrate fruits such as berries. We instruct participants to avoid sugar-sweetened foods (eg, sugar-laden desserts such as cakes, cookies, and ice cream), sugar-sweetened beverages (eg, sugared sodas and sweetened teas and coffees), naturally sweet foods (eg, tropical fruits), and starchy foods (eg, foods made with grain-based flours such as bread, pasta, tortillas, breakfast cereals, and pastas, as well as potatoes and rice). Each week, we provide class participants with CR diet recipes and a lending library of CR diet cookbooks (see Table 5 for core and booster diet curriculum components or weekly topics).

#### Teacher

A board-certified internal medicine physician who has transitioned patients with T2DM onto CR diets in her private practice is the primary teacher who leads all diet classes.

#### Fidelity Check

We record all class sessions, and a PhD-level clinical health psychologist and a PhD-level social health psychologist, the latter of whom originally developed the diet classes [8], review a randomly selected subset of these recordings to ensure that the diet class does not include content related to mindful eating. The primary teacher meets with one or both psychologists weekly by phone to review events occurring during classes and to review material for the following week. When the primary teacher is unavailable to teach (on rare occasions), one of the psychologists serves as a substitute. Study psychologists observe one-sixth of the CR classes in person.

### Mindful Eating Intervention

We deliver the Eat Right Now (ERN) [26] program, which is a mindful eating intervention in the form of a mobile app. It functions on both iOS and Android platforms, and we administer it in combination with a weekly 1-hour group class.

**Table 5:** Core and booster diet curriculum components.

Session	Topics
Week 1	Introduction to the program; information about insulin, diet, and type 2 diabetes; overview of previous research on very low-carbohydrate diets for adults with type 2 diabetes; what to eat on a very low-carbohydrate diet; how to count net (nonfiber) grams of carbohydrates; sample shopping list; example breakfast and snacks; how to deal with potential side effects of the diet; encouragement to change breakfasts and snacks to be very low-carbohydrate.
Week 2	The history of using a very low-carbohydrate diet for type 2 diabetes; very low-carbohydrate lunch suggestions; ideas for eating at restaurants; suggestions for planning ahead; list of low-carbohydrate vegetables; encouragement to clear noncompliant foods out of their pantries; suggestion to add very low-carbohydrate lunches to their already very low-carbohydrate breakfasts and snacks.
Week 3	Overview of the increase in type 2 diabetes over time; very low-carbohydrate dinner suggestions; encouragement to pick out recipes from cookbooks and online resources; suggestion to keep a favorite foods diary in order to track very low-carbohydrate foods they enjoy; information about non-nutritive sweeteners and sugar alcohols; suggestion to add very low-carbohydrate dinners.
Week 4	The meaning of and how to measure blood ketone levels; how to deal with potential side effects of being in nutritional ketosis; inspirational stories from others trying this approach to help manage their type 2 diabetes.
Week 5	Discussion of participants’ perception of testing for ketones over the previous week; discussion about types of fat and encouragement to add fat to their diet; types of lower carbohydrate fruits; the benefits of sleep and information about sleep hygiene.
Week 6	Ways to break through a weight-loss plateau; encouragement to increase food variety; information about alcohol.
Week 7	Very low-carbohydrate travel suggestions; fast food options; snack ideas; problem-solving tips for eating on the meal plan.
Week 8	Coping with peer pressure to not comply with the meal plan; suggestions for very low-carbohydrate party and holiday food; encouragement to safely add in physical activity; information about coping with physical challenges related to physical activity.
Week 9	Reminders of the diet basics; suggestion to pay attention to food sensitivities; information about cholesterol types and health risk; description of the health impact of sugar consumption; reminders for how to order very low-carbohydrate meals at restaurants.
Week 10	Discussion of example restaurant menus; self-assessment of their dietary adherence (technical, psychological, or external struggles).
Week 11	Information about changes in hunger and flavor when following a very low-carbohydrate diet; partnered sharing of the program so far.
Week 12	Ways to recover from slips and stick to their program-related goals; reminders of tricky dietary issues.
Week 16 (Maintenance)	Success story of a physician treating his own and others’ type 2 diabetes using a very low-carbohydrate diet; how to access recorded presentations given by physicians about using a very low-carbohydrate diet for type 2 diabetes (freely available online); information about how a very low-carbohydrate diet has been used to help other health conditions.
Week 20 (Maintenance)	How fruits and vegetables have changed over time; information about online support groups; how to cope with being under the weather/ill and following a very low-carbohydrate meal plan.
Week 24 (Maintenance)	Case studies of several long-term adherents to a very low-carbohydrate diet; description of research on the long-term impact of a very low-carbohydrate diet for type 2 diabetes; suggestions for fine-tuning the diet to potentially reduce red and processed meat consumption; encouragement to stick with the program.

#### Mobile App

This 28-module course has been tested in overweight women who report problematic overeating in response to food cravings [26]. Participants in that study experienced reductions in daily craving-related eating (assessed using EMA), as well as reductions in trait-level measures of overeating, including reward-based eating drive, eating to cope with negative emotions, and food cravings. ERN intervention components center on (1) the scientific underpinnings of how food cravings arise and are reinforced, (2) research on the behavioral conditioning processes by which responses to food cravings become habitual, and (3) research showing how mindfulness directly targets cravings to change behavior. ERN modules teach participants to attend to three aspects of eating: why, what, and how: *Why* they eat, including environmental and emotional triggers unrelated to homeostatic hunger; *What* types of food are most likely to lead to and reinforce cravings; and *How* to eat with awareness and mindful attention to physiological cues. Each module lasts approximately 5 to 10 minutes, and participants can only access one new module per day (after completing the previous module). Participants have unlimited access to previous modules. We ask participants to complete 2 or 3 modules per week during the intervention period, which translates to about 1 module every 2 days. In addition to the modules, participants can access extra tools to aid in mindfully “riding out” food cravings as they occur. Each of these tools targets disruptions in automatic, “mindless” eating in response to cravings and emphasizes attending to physiologic hunger signals. Participants can set reminders within the app that encourage them to check in with their hunger and emotional state and to use mindfulness skills that help cultivate mindful eating habits. After the 28 modules are complete, all tools and previous modules remain available for the duration of the trial to support ongoing mindful eating and skill use (see Textbox 2 for a description of intervention content and [26] for further details about the ERN intervention).

#### Weekly Classes

Each in-person weekly class focuses on participants’ experiences in learning and applying mindful eating skills that they learn from the modules. Weekly classes are not didactic in nature but rather center around discussing participants’ experiences with mindful eating practices, troubleshooting obstacles, and engaging in and reflecting on group exercises. If randomized to the mindfulness treatment arm, participants attend this class in the hour before attending the CR diet class. If they wish, participants can arrive 30 minutes early to class to watch an ERN module of their choice as a group.

#### Teachers

Two mindful eating teachers colead most mindful eating classes, with a single teacher leading the session when the second teacher is not available. Both teachers completed supervised training in the ERN intervention according to a standardized teacher training process led by Dr Judson Brewer, MD, PhD, who developed the ERN mobile intervention.

#### Fidelity Check

We audio record all mindful eating classes. An instructor in the Mindful Eating Facilitator Training course reviews a randomly selected subset of these recordings and conducts supervision calls with study teachers. During these calls, the instructor provides feedback on adherence to the ERN model, teaching style, and group facilitation processes. Teachers are aware of the content in the diet classes, and we instruct them to avoid messages that might conflict with the diet class content.

### Interventions: Maintenance Phase

After the weekly intervention phase (12 weeks), participants receive the following maintenance support:

### Monthly Diet Booster Classes

During monthly diet booster classes, the teacher provides supplemental material intended to troubleshoot problems, discusses progress and barriers to progress, and engages participants in motivating conversations geared toward continuation of assigned intervention-related behaviors. Monthly class content features information about how physicians are using CR diets to help their patients with T2DM and other metabolic conditions, the experiences of long-term adherents to a CR diet, and other related topics (see Table 6 for additional detail).

### Weekly Diet Individual Meetings

During these brief (15 min) individual meetings, which are held over the phone, the diet teacher answers any CR diet-related questions and concerns that may be individually specific to the participant. These individual meetings allow teaching to be tailored to individual needs related to several diet-related topics, such as weight loss stalls, lack of food variety, difficulties remaining in ketosis, or poor dietary adherence. The individual meetings are only offered to select participants. Over the course of the 3 planned waves, we test methods of selecting participants for these individual consultations, including the feasibility and acceptability of offering the individual consultations to a subset of participants based on ketone levels at 12 weeks.

**Mindful eating training components.**

Modules 1-7: Goals, habit formation, body scan, behavioral management, self-monitoring, existing with cravings

- Modules 1 and 2 allow the participant to set goals and teach them how habits form (eg, positive and negative reinforcement). These modules also introduce the roles of self-monitoring and mindless eating. Module 3 introduces basic mindfulness practices such as the body scan, which targets bodily awareness and momentary concentration. Module 4 defines refined, hyper-processed, calorically dense foods, with an emphasis on how these foods can have addictive qualities. It also defines healthier, nutrient-dense, lower-glycemic foods. Module 5 teaches how to mindfully work with food cues, affective states, and food cravings using the Recognize, Allow, Investigate, Notice (RAIN) exercise. This exercise promotes the dissociation between experiencing food cravings and eating in response to them. Module 6 helps individuals recognize self-judgment and how to be kind to themselves if they feel that they have “screwed up.”

Modules 8-14: Noting practice, barriers to change, concept reinforcement

- Modules 8 through 14 emphasize the use of noting practice (the “N” of RAIN). Modules in week 2 encourage participants to note what their hunger, craving, and satiety levels are while eating and when considering eating. Participants view several animations that reinforce their understanding of how people “feed” their cravings by eating.

Modules 15-21: Loving-kindness, curiosity

- Modules 15 through 21 continue to reinforce noting practice and teach users to differentiate emotional eating (eg, due to stress) from physiological, homeostatic hunger (module 15). Modules 16 and 17 reinforce self-kindness (eg, loving-kindness) and curiosity, respectively.

Modules 22-28: Strategizing, reinforcing, next steps

- Modules 22 through 28 introduce how habitual thought patterns can be obstacles, and how we can mindfully observe our thoughts. Week 4 modules also help individuals reflect on their own experience from the previous few weeks of changing their relationship to food cravings. These reflections facilitate participants’ awareness of their new habits and a gradual shift away from experiencing (and indulging) food cravings without awareness toward mindfully attending to their food craving triggers. Week 4 gives participants a sense of which modules they might want to review to reinforce certain lessons or tools (eg, excessive self-judgment).

**Table 6:** Schedule and time commitments of intervention activities.

Phase and activity			Activity schedule		Total time commitment		CR^a^-only group		CR and mindful eating group
**Intervention phase (weeks 1-12)**									
	Diet classes	Weekly for 1 hour		12 hours		✓		✓	
	Mindful eating classes	Weekly for 1 hour		12 hours		—		✓	
	Eat Right Now app	Engage 2 or 3 times per week for 5-10 min		4 hours for required videos; additional time using app as desires		—		✓	
**Maintenance phase (weeks 13-24)**									
	Diet booster classes	Monthly for 1 hour		3 hours		✓		✓	
	Diet individual meetings (15 min by phone)^b^	Up to weekly, but typically biweekly or less often		0-3 hours		✓		✓	
	Mindful eating booster classes	Monthly for 1 hour		3 hours		—		✓	
	Mindful eating Zoom sessions^c^	Weekly for 1 hour, no meetings week of booster classes		0-8 hours		—		✓	
Total Time			18 required hours		37 required hours + 13 optional hours		—		—

^a^CR: carbohydrate-restricted.

^b^Encouraged but optional; offered to select participants who schedule as they wish up to weekly.

^c^Encouraged but optional.

### Mindful Eating Booster Classes and Weekly Group Internet-Video Meetings (Zoom)

The mindful eating booster classes follow the same interactive format as the in-person weekly classes conducted during the intervention phase. During the intervention phase, these classes are held in-person once a month. During the maintenance phase, these classes are held over Zoom videoconferencing on weeks in which no in-person class is held. During the maintenance phase, in both the monthly in-person classes and the weekly Zoom classes, teachers continue to deliver the ERN intervention and focus on questions and challenges that participants bring up during class. Though we assign modules in-between all classes held during the intervention phase, we do not assign modules during the maintenance phase, as participants will have already completed the full 28-day curriculum.

### Safety Monitoring

#### Glucose Monitoring and Medication Adjustment

CR diets often lead to decreases in glucose levels in people with T2DM. This increases the risk of hypoglycemia among individuals using glucose-lowering medications, such as sulfonylureas or insulin, unless appropriate medication adjustments are made. In our previous research [8], we used a medication reduction protocol designed to reduce the risk of hypoglycemia when initiating a CR diet, which resulted in no episodes of clinically significant hypoglycemia. For this trial, we use a similar protocol. Study physicians (medical doctors) who have experience in transitioning people with T2DM onto low-carbohydrate diets (one internal medicine physician, one endocrinologist physician) will serve as study physicians. These study physicians review participants’ medications before participants begin the nutritional intervention. Study physicians oversee changes in medications, specifically reductions (or discontinuations if at a low dose) in an order that typically prioritizes reductions/discontinuations as follows: (1) insulin, (2) sulfonylureas, (3) meglitinides (secretagogues), (4) SGLT-2 inhibitors, (5) GLP-1 agonists and DPP4 inhibitors, and (6) thiazolidinediones (TZD). Biguanides (eg, metformin) are typically continued unless glycosylated hemoglobin levels (HbA_1c_) are consistently below 6.5%. Initial medication adjustments typically involve reducing a medication dose by half and are aimed at preventing hypoglycemia when initiating carbohydrate restriction. The number of medications reduced is calibrated based on initial HbA_1c_ levels, with limited medication reductions in participants with high HbA_1c_ levels and more extensive reductions in participants with HbA_1c_ levels in the low diabetic range. For example, a participant with an HbA_1c_ level greater than 8.0% might have a recommendation to reduce insulin doses in half while continuing other medications, while a participant with an HbA_1c_ level between 6.5% and 7.0% might have a recommendation to stop using all diabetes medications other than biguanides and TZDs. We ask participants using all glucose-lowering medications other than metformin to measure their blood glucose at least once daily (before dinner) to ensure they are not at risk for hypoglycemia. For participants who are on a regimen considered to have additional risks of hypoglycemia (eg, insulin), we ask them to also measure their blood glucose in a fasted state (before breakfast). We review symptoms of hypoglycemia with participants in the initial group meeting and provide the following instructions: any participant who believes that he or she has hypoglycemia should immediately check his or her glucose level (fingerstick), if possible, and should consume carbohydrates if their glucose levels are below 70 mg/dL. If a glucose level cannot be obtained promptly, they should consume carbohydrates. We instruct all participants to report hypoglycemic symptoms to study staff. Study staff notify study physicians immediately upon learning that a participant has reported hypoglycemic symptoms or glucose levels below 80 mg/dL if on medications other than metformin; hypoglycemic symptoms or glucose levels below 60 mg/dL if using either metformin alone or no glucose-lowering medications; or glucose levels above 220 mg/dL (all participants). Participants can contact study staff by phone call, text message, or email to ask questions and report symptoms or problems, and all study staff can then immediately reach study physicians by mobile phone or pager for urgent problems. Study staff collect regular glucose information from participants weekly (online, Qualtrics). Study physicians review glucose values at least weekly and make medication adjustments based on glucose levels, current medication regimens, and how long the participant has followed the CR diet. Predinner glucose levels below 110 mg/dL typically trigger evaluation of further medication reductions, using the priority list described above, in participants receiving medications other than biguanides. We provide participants’ primary care physicians with information about the study before participants begin the study, notify them of any changes to medication regimens, and consult with them as needed.

#### Hyperlipidemia

Some clinical trials of CR diets for obesity have demonstrated hyperlipidemia in response to a high-fat diet; hence, we will monitor participants for this possibility by testing serum lipids at baseline as well as at 12 and 24 weeks. Lipid studies include lipid particle size assessment using nuclear magnetic resonance methods (LabCorp, NMR Lipoprofile [34]). Although many people tolerate increases in saturated fat without deleterious changes in blood cholesterol levels on a CR diet, a minority experience increases in low-density lipoprotein (LDL) cholesterol. Participants with significant increases in LDL cholesterol, particularly increases accompanied by an increase in small particle LDL (small particle LDL cholesterol being the most strongly linked to increases in cardiovascular disease risk), receive counseling on how to follow a CR diet that limits saturated fat and increases monounsaturated and polyunsaturated fats, such as those found in olive oil and nuts.

#### Minor Side Effects

Minor side effects including constipation, headache, diarrhea, muscle cramping, rash, or general weakness may occur when initiating a CR diet. Most side effects occur at the beginning of the diet transition, are short-lived, and are generally well-treated with adjustments to fluid intake and other diet modifications that are thoroughly addressed at the first diet class and throughout the study. We monitor for and record side effects, and study staff involve the diet teacher and study physicians as needed.

#### Data Safety Monitoring Board (DSMB)

We have created a 2-person DSMB. We hold annual DSMB meetings via Zoom videoconference. In the case of a serious adverse event (SAE), we will call for a special closed meeting of the DSMB to review any needed changes or early stopping of the trial (see 2.10.8, Interim Analyses, Stopping Rules). In this trial, an adverse event (AE) is defined as any unfavorable and unintended diagnosis, symptom, sign (including an abnormal laboratory finding), syndrome or disease which either occurs during the study, having been absent at baseline, or if present at baseline, appears to worsen. AEs will be recorded regardless of their relationship to the study intervention. An SAE is defined as any untoward medical occurrence that results in death, is life-threatening, requires inpatient hospitalization or prolongation of existing hospitalization, results in persistent or significant disability/incapacity, or is a congenital anomaly.

AEs that are collected as solicited events at study visits include recent hospitalizations or new medical diagnoses or problems. We will also record unsolicited events reported by participants. AE data that are formally assessed at study visits will be compared with existing unsolicited events in participant records to avoid double capture. The study team reviews all potential AEs reported by study participants and determines their relatedness to diet or study intervention, expectedness, and severity.

We monitor hypoglycemia and serum fasting lipid profiles. As shown in Table 7, we use the Common Terminology Criteria for Adverse Events (CTCAE) as follows, with grades 1 to 3 considered AEs, and grades 4 and 5 considered SAEs.

### Statistical Methods

#### Preliminary Analyses

We will examine data distributions at baseline using summary statistics and will use graphical methods to visualize data to identify possible outliers and non-normal distributions before proceeding with modeling. Discussions of outliers and of missing data are included below (see “Sensitivity Analyses” and “Missing Data”).

**Table 7:** Criteria for adverse events.

CTCAE^a^ term	Grade 1	Grade 2	Grade 3	Grade 4	Grade 5
Cholesterol high	>ULN^b^-300 mg/dL	>300-400 mg/dL	>400-500 mg/dL	>500 mg/dL	—
Hypertriglyceridemia	150 mg/dL-300 mg/dL	>300 mg/dL-500 mg/dL	>500 mg/dL-1000 mg/dL	>1000 mg/dL; life-threatening consequences	Death
Hypoglycemia	<LLN^c^-55 mg/dL	<55-40 mg/dL	<40-30 mg/dL	<30 mg/dL life-threatening consequences; seizures	Death

^a^CTCAE: Common Terminology Criteria for Adverse Events. CTCAE criteria available [66].

^b^ULN: upper limit of normal.

^c^LLN: lower limit of normal.

**Table 8:** Primary and secondary trial outcomes.

Measure	Construct assessed	Primary outcome	Secondary outcome	Outcome type
Ecological momentary assessment of eating behavior	Craving-related eating	✓	—	Behavioral
Computerized cognitive assessment: delayed discounting	Impulsivity	—	✓	Behavioral
Palatable Eating Motives Scale: Coping Subscale	Stress-related eating	—	✓	Behavioral
24-hour dietary recall	Dietary adherence	—	✓	Behavioral
Blood ketone levels	Dietary adherence; dietary adherence after nonadherence	—	✓	Clinical
Weight	Weight	—	✓	Clinical
Glycosylated hemoglobin A_1c_ (HbA_1c_)	Glycemic control	—	✓	Clinical
Glucose, plasma	Glycemic control	—	✓	Clinical
Insulin, plasma	Glycemic control	—	✓	Clinical

#### Primary Outcome

As shown in Table 8, the primary outcome is reduction in craving-related eating, as measured through EMA text messages. Participants respond to the EMA question, “Did you eat or drink anything in response to that craving?” as *yes* or *no* (see Table 3). We will use a mixed effects logistic regression model, with random effects for day nested within study period nested within person, and for each of up to 4 possible reports per day (morning, afternoon, evening, and “any other time not previously reported”), to predict the dichotomous primary outcome (yes/no responses to EMA question). Model covariates (fixed effects) include time of day, study period, randomization group, and the interaction of study period by randomization group. This will allow us to estimate the odds of indulging a craving at 3 and 6 months compared with baseline, separately for each arm, as well as allowing for direct comparisons between study arms at each time.

#### Secondary Outcomes

Secondary outcomes include changes in impulsivity, stress- and emotion-related eating, glycemic control (HbA_1c_, fasting glucose, and insulin resistance), and weight. We also assess dietary adherence (ketone levels) as a secondary outcome (see Table 8 for all secondary outcome variable descriptions).

To assess changes in impulsivity, stress- and emotion-related eating, glycemic control, and weight (all treated as continuous variables), across groups and over time, we will use ANCOVA and linear mixed methods. In mixed models, we will include a random person effect, as well as fixed effects including study period, randomization group, and their interaction term. We will estimate differences between randomization groups at each time point and differences between time points within each randomization group.

To assess dietary adherence (ketone levels) and resilience following dietary nonadherence, we will first dichotomize ketone data as “ketosis” (values ≥0.3 BOHB mmol/L in fingerstick blood) and “not ketosis” (values <0.3 BOHB mmol/L in fingerstick blood). We have dichotomized ketone levels based on our use of this level as an indicator that participants are engaging in adequate CR to achieve nutritional ketosis.

To assess overall dietary adherence, we will use a mixed effects logistic regression model with random effects for study period nested within person, as each participant provides ketone data 3 times per week for several weeks within each study period. We will include (as fixed effects) study period, randomization group, and their interaction term. We will estimate differences between randomization groups at each time point and differences between time points within each randomization group. We will also explore models treating ketone data continuously using the same model specifications as described above. This will yield average differences in the ketone levels between randomization groups and within time points.

To assess resilience following dietary nonadherence, we will compare the amount of time that passes between obtaining a ketone value <0.3 BOHB mmol/L (not in ketosis) after having had a prior ketone value of ≥0.3 BOHB mmol/L (in ketosis) between study groups, using a repeated measures analysis to address multiple episodes within participants, if these occur.

#### Sensitivity Analyses

If outliers are present in continuous measures, we will rerun models after Winsorizing these data using 3 SDs from the mean cut-off to minimize the impact of extreme values [67].

#### Missing Data

An important goal is to achieve high levels of participant retention and to minimize missing data. When consenting and enrolling participants, we emphasize the importance of completing study assessments and explain that missing data negatively impact scientific research. We provide financial compensation for the time participants spend completing study measures, and we devote significant staff resources to allow for careful participant follow-up. Mixed effects models using maximum likelihood (ML) estimation limit the risk of biased results due to missing data [68]. Our analytic approach uses these methods to minimize the impact of missing data.

**Table 9:** Power to detect between-group differences based on one-sided and two-sided tests for a difference in means between groups.

Power for a one-sided test	Power for a two-sided test	Alpha	Control arm (n)	Mindful eating arm (n)	Days with craving-related eating in control arm (%)	Days with craving-related eating in mindful eating arm (%)	Delta (difference between arms)	SD
0.7855	0.6786	.05	30	30	50	43	−7	11
0.8726	0.7910	.05	30	30	50	42	−8	11
0.7855	0.6786	.05	30	30	49	42	−7	11
0.8726	0.7910	.05	30	30	49	41	−8	11
0.7855	0.6786	.05	30	30	48	41	−7	11
0.8726	0.7910	.05	30	30	48	40	−8	11
0.7855	0.6786	.05	30	30	47	40	−7	11
0.8726	0.7910	.05	30	30	47	39	−7	11

#### Data Management

Data are collected by trained research assistants and study coordinators using paper forms and Web-based questionnaires (online, Qualtrics). All surveys and forms are deidentified and coded with a unique subject number.

All data collection forms and laboratory reports are reviewed by the study team and data entry staff, who ensure that they are accurate and complete. A person who has not collected or entered data reviews the entered data for quality assurance. Protocol compliance and monitoring is done via review of records and forms after each participant visit and before data entry. Review is done by a study staff member who is not involved in the data collection for that participant, and any discrepancies or potential problems are reviewed by the Project Director or a senior study coordinator. Protocol deviations are captured by regular review of cases during the enrollment process by the Project Director to ensure that eligibility criteria are met. Key points of review include postconsent visit and baseline lab completion before randomization.

#### Sample Size Estimates

Although this study is intended to provide a foundation for future trials of mindfulness and dietary change and the primary focus is not on having adequate power for hypothesis testing, we have performed a statistical power analysis for our primary outcome, which is craving-related eating.

Our preliminary data showed that participants experienced eating in response to a craving on 50% of days at baseline (SD 11%). We aim to assess whether participants who receive the mindful eating intervention experience a decrease in craving-related eating relative to participants who do not receive the mindful eating intervention. Because we anticipate a directionality of effect (we hypothesize greater adherence over time among the participants who receive the mindful eating training), we planned our sample size around a one-sided test but also performed a power analysis using a two-sided test. We estimate that we will have greater than 80% power to detect a difference between study groups if there is a between-group difference in craving-related eating at follow-up of 8 percentage points using a one-sided test and 79% power using a two-sided test (see Table 9).

#### Interim Analyses, Stopping Rules

We have not planned any interim analyses, and we do not anticipate stopping the study early. Although our main clinical outcome of interest, HbA_1c_, is a biomarker indicating long-term risk of clinical events due to diabetes (eg, neuropathy, blindness), it is not an outcome that would justify early stopping rules for a trial of this size and duration. The DSMB will review any SAEs related to the intervention or assessment procedures in separately scheduled, closed meetings (if SAEs were to occur) to determine whether the study should be stopped. The DSMB chair will moderate any closed sessions and collect a formal vote from DSMB members as to whether the trial should continue given the occurrence of an AE.

## Results

The project was funded in September of 2016 and final classes ended in March of 2018. Data analysis is currently underway, and we expect to submit results for publication in early 2019.

## Discussion

### Principal Findings

Adhering to dietary recommendations poses challenges for a variety of conditions, including obesity and diabetes [16,21]. This intervention is based upon a model that postulates that for many people, it is difficult to adaptively respond to food cravings in ways that maintain adherence to a dietary prescription. Although previous mindfulness-based interventions have begun to suggest that mindfulness-based interventions may impact obesity-related behaviors (yet, not necessarily weight loss itself) [69-71], we are employing a specific component of mindfulness (ie, mindful eating), which we hypothesize to more specifically impact obesity-related behaviors.

Prior work has documented that decreasing carbohydrate intake in people with T2DM leads to improved glycemic control [14] and that following a low-carbohydrate diet can reduce cravings for carbohydrate-rich foods [23]. Despite this “best case” scenario for limiting cravings for carbohydrate-rich foods, we postulate that adherence to carbohydrate restriction can be challenging for many people because of difficulty coping with persistent food cravings. The modern food environment facilitates the experience of food cravings and also ensures that inexpensive food to satisfy these cravings is readily available. Prior data on the importance of food cravings in adherence to nutrition recommendations in T2DM leaves some uncertainties. Some studies have reported increases in food cravings during weight loss diets [72], but this type of study does not address persons with T2DM, and the focus on calorie restriction as opposed to diet pattern also differs from this study. Of more direct relevance, a recent study of persons with T2DM has reported that, in general, persons with T2DM put on a weight loss diet with differing proportions of protein and carbohydrate reported reductions in food cravings, which did not differ by diet composition [73]. The overall reduction in food cravings was moderate, however, with a 15% improvement in one measure of food craving, indicating that while cravings improved, they did not go away. Weight was positively correlated with cravings. The authors concluded that “Reductions in the frequency of food cravings and improvements in eating behaviors may encourage compliance and adherence to lifestyle programs which ultimately may enhance diabetes management.” Given some uncertainties in existing literature, our study is aimed, in part, at clarifying the role that reductions in eating in response to food cravings may play in improving nutritional adherence in T2DM.

In addition to addressing eating in response to food cravings, we also postulate that other behavioral pathways may be relevant in improving diet adherence in T2DM. For example, resuming dietary adherence following lapses in adherence poses a significant challenge and remains understudied [74]. Our study directly tests mindful eating training as an intervention to strengthen adaptive coping with food-craving experiences and to resume dietary adherence following lapses.

Although we ultimately aim to impact clinical outcomes (eg, HbA_1c_), this trial differs from other trials by focusing on behavioral mechanisms through which our intervention may impact clinical outcomes. Thus, our hypothesized behavioral mechanisms are the primary endpoints at this stage of our research. This study is therefore aligned with models advocated by the Science of Behavior Change (SOBC) model [75] and the Obesity-Related Behavioral Intervention Trials (ORBIT) model [76]. These models advocate identifying and individually testing key mechanisms that may underlie successful behavior changes and then developing and testing interventions that target these mechanisms. These models focus on maximizing potential to optimize intervention potency, efficiency, and effectiveness.

This trial includes several innovative features. First, it focuses on identifying mechanisms that underpin behavioral factors that influence health outcomes, that is, ascertaining if increasing training in mindful eating (mechanism) can increase dietary adherence (behavioral factor) holds implications for dietary interventions in T2DM (clinical health outcomes). Dietary prescriptions can effectively improve glycemic control to the extent that they are followed [13]; hence, developing methods to increase dietary adherence is a critical public health need. Second, this trial focuses on testing these mechanisms, in part, using a cost-effective hybrid method that can be implemented in a variety of health care settings, including those with greater limitations on in-person services, that is, the didactic portion of the mindful eating training being tested in this trial is currently available for health care providers to prescribe to patients with smartphones, which 77% of US adults own [77]. The smartphone-based administration method ensures that all recipients of this intervention receive the intervention exactly as it was tested (ie, no effects of different intervention instructors). Mobile platforms allow patients to integrate behavioral change components as they go about their daily lives [78]. Third, this trial uses EMA in the form of mobile text messaging, which reduces issues associated with recall [79]. These ambulatory measures are important for both study providers and recipients. Such measures provide behavioral feedback to study staff, who can engage with patients to identify problems (both for intervention development purposes and to assist the patient with solutions), as well as provide actionable feedback directly to the patients, who can self-correct based on data they collect. Fourth, this trial uses both biological (blood ketone testing) and self-report (24-hour dietary recall) methods to assess dietary adherence.

### Limitations

Limitations of this trial include that it is powered to examine behavioral pathways we hypothesize to be tied to clinical outcomes rather than to evaluate clinical outcomes themselves, such as improvements in glycemic control. As described in the ORBIT model for developing behavioral treatments for chronic diseases [76], this trial is situated at phase IIb, which tests for a signal in the behavioral target while using a control group. Previous research has found that greater levels of mindfulness are associated with less stress- and emotion-related eating [29] and suggests that completing the mindfulness intervention administered in this trial leads to reductions in stress- and emotion-related eating, craving intensity, and rate of eating in response to cravings [26]. The next phase of this work will include a larger sample size that will provide better statistical power to examine clinical outcomes. Additionally, future work should test strategies for maintenance of behavioral (and clinical) changes. Moreover, our trial design and control group, although informed by the ORBIT model, does leave open the possibility that the additional content provided to the mindful eating group (eg, mobile app, more class time) may also influence our outcomes above and beyond the mindful eating instruction, as the groups may have differed in terms of time, attention from teachers, and participants’ expectations.

We designed this two-group randomized controlled trial to test behavioral pathways through which mindfulness may impact the ability to respond to food cravings in ways that are consistent with a dietary prescription for T2DM. The findings may be relevant for understanding the effects of mindfulness interventions on dietary adherence in the context of diabetes, obesity, and other chronic metabolic diseases.

## Appendix

Multimedia Appendix 1 Peer-reviewer report from the NIH.

## Abbreviations

AE: adverse event
BMI: body mass index
BOHB: β-hydroxybutyrate
CR: carbohydrate-restricted
DSMB: Data Safety Monitoring Board
EMA: ecological momentary assessment
ERN: Eat Right Now
HbA_1c_: glycosylated hemoglobin
hsCRP: high-sensitivity C-reactive protein
IRB: institutional review board
LDL: low-density lipoprotein
ML: maximum likelihood
NCCIH: National Center for Complementary and Integrative Health
NDSR: Nutrition Data System for Research
NIH: National Institutes of Health
ORBIT: Obesity-Related Behavioral Intervention Trials
SAE: serious adverse event
SOBC: Science of Behavior Change
T2DM: type 2 diabetes mellitus
TSH: thyroid stimulating hormone
TZD: thiazolidinediones
UCSF: University of California, San Francisco
