# Correction: Examining the Effects of Mindful Eating Training on Adherence to a Carbohydrate-Restricted Diet in Patients With Type 2 Diabetes (the DELISH Study): Protocol for a Randomized Controlled Trial

**DOI:** 10.2196/17226

**Published:** 2020-01-13

**Authors:** Ashley E Mason, Laura Saslow, Patricia J Moran, Sarah Kim, Priyanka K Wali, Hiba Abousleiman, Alison Hartman, Robert Richler, Samantha Schleicher, Wendy Hartogensis, Elissa S Epel, Frederick Hecht

**Affiliations:** 1 UCSF Osher Center for Integrative Medicine Department of Medicine University of California San Francisco San Francisco, CA United States; 2 Center for Health and Community Department of Psychiatry University of California San Francisco San Francisco, CA United States; 3 School of Nursing Department of Health Behavior and Biological Sciences University of Michigan Ann Arbor, MI United States; 4 Division of Endocrinology, Diabetes and Metabolism Department of Medicine San Francisco General Hospital San Francisco, CA United States; 5 School of Medicine University of Maryland Baltimore, MD United States

The authors of “Examining the Effects of Mindful Eating Training on Adherence to a Carbohydrate-Restricted Diet in Patients With Type 2 Diabetes (the DELISH Study): Protocol for a Randomized Controlled Trial” (JMIR Res Protoc 2019;8(2):e11002), noticed two errors in the author information of their publication.

Priyanka K Wali, MD, was inadvertently omitted from the author list of the manuscript. Dr Wali’s name has now been included as the fifth author of the paper, between authors Sarah Kim and Hiba Abousleiman. Dr Wali’s affiliation is:

UCSF Osher Center for Integrative Medicine, Department of Medicine, University of California San Francisco, San Francisco, CA, United States

Furthermore, the affiliation for author Laura Saslow was incorrectly listed in the original published manuscript as:

UCSF Osher Center for Integrative Medicine, Department of Medicine, University of California San Francisco, San Francisco, CA, United States

Laura Saslow's correct affiliation is:

School of Nursing, Department of Health Behavior and Biological Sciences, University of Michigan, Ann Arbor, MI, United States

These changes do not impact the Acknowledgments or Conflicts of Interest statement, nor do they impact the equal contribution footnote.

The corrections will appear in the online version of the paper on the JMIR website on January 13, 2020, together with the publication of this correction notice. Because this was made after submission to PubMed, PubMed Central, and other full-text repositories, the corrected article has also been resubmitted to those repositories.

