# Supplementary material for: Examining the Effects of Mindful Eating Training on Adherence to a Carbohydrate-Restricted Diet in Patients With Type 2 Diabetes (the DELISH Study): Protocol for a Randomized Controlled Trial
Source: JMIR Res Protoc. 2019 Feb 20;8(2):e11002. doi: 10.2196/11002 (PMC6401674; doi:10.2196/11002)
Supplement: Multimedia Appendix 1 [file resprot_v8i2e11002_app1.pdf]

SUMMARY STATEMENT

PROGRAM CONTACT:
Dr. WEN CHEN
301-451-3989
chenw@mail.nih.gov

(Privileged Communication)

Release Date: 04/24/2016 10:22 AM
Revised Date:

Application Number: 1 R61 AT009333-01

Principal Investigators (Listed Alphabetically):

EPEL, ELISSA S.
HECHT, FREDERICK M (Contact)

Applicant Organization: UNIVERSITY OF CALIFORNIA, SAN FRANCISCO

Review Group: ZAT1 SM (41)
National Center for Complementary and Integrative Health Special Emphasis Panel
Mind and Body Interventions

Meeting Date: 04/08/2016
Council: MAY 2016
Requested Start: 07/01/2016
RFA/PA: AT16-005
PCC: CHENW

Project Title: Optimizing lifestyle interventions with mindfulness-based strategies in type 2 diabetes
SRG Action: Impact Score:37
Next Steps: Visit http://grants.nih.gov/grants/next_steps.htm
Human Subjects: 30-Human subjects involved - Certified, no SRG concerns
Animal Subjects: 10-No live vertebrate animals involved for competing appl.
Gender: 1A-Both genders, scientifically acceptable
Minority: 1A-Minorities and non-minorities, scientifically acceptable
Children: 1A-Both Children and Adults, scientifically acceptable
Clinical Research - not NIH-defined Phase III Trial

Project Year	Direct Costs Requested	Estimated Total Cost
1	274,288	367,546
2	274,640	368,018
3	499,310	669,075
4	499,880	669,839
5	499,294	669,054
TOTAL	2,047,412	2,743,532

1R61AT009333-01 HECHT, FREDERICK

RESUME AND SUMMARY OF DISCUSSION: This new R61/R33 application “Optimizing lifestyle interventions with mindfulness-based strategies in type 2 diabetes” is submitted in response to RFA-AT-16-005 “Phased Innovation Award for Mechanistic Studies to Optimize Mind and Body Interventions in NCCIH High Priority Research Topics (R61/R33)” by The Regents of the University of California, San Francisco; Department of Medicine, with Frederick Hecht MD and Elissa Epel PhD as the principal investigators. The applicants are proposing to test the hypothesis that improved ability to manage food cravings and emotional eating is a key mechanism through which mindfulness-enhancements can improve dietary adherence. The R61 phase will test the “ability to adaptively experience food cravings” as the central mechanism whereby mindfulness skills enhance dietary adherence. The R33 phase will further optimize the mechanism through the use of an adaptive intervention strategy. Type 2 diabetes and obesity are important public health problems with rising prevalence. There is a great potential for non-medication treatments like lifestyle interventions to positively impact T2D. If mindfulness is effective for enhancing the capacity to resist food cravings results may be generalizable to the management of addiction which is also characterized by craving. The use of a mindfulness-based intervention to target stress generally and food cravings specifically is novel and could have broad applicability. Use of ecological momentary assessment to measure cravings and stress triggered eating is innovative. The use of ketone monitoring as an adherence and biomarker outcome across treatment interventions is interesting. There is enthusiasm for the use of an adaptive intervention design to test the different maintenance approaches. The EMA data collection procedure is intriguing. The principal investigators are well suited to conduct the proposed study. They have a complimentary set of experience and a history of effective collaboration. They have assembled a highly qualified team of collaborators. The leadership approach, governance and organizational structure are appropriate. An outstanding environment and excellent resources will be available. The overall strategy, methods and analyses are well-reasoned and appropriate to accomplish the specific aims. Alternative strategies and potential pitfalls are considered. Pairing of subjective measures of craving with objective measures of behavior as primary outcomes tested across time and between groups is a robust way to evaluate the proposed mechanism. Outcomes are generally well described, sufficiently justified and positioned to provide interpretable results. Additional measurements with delayed-discounting task and self-report questionnaires further enhance the depth and breadth of information. Recruitment and retention plans are adequately described. Go/No-Go criteria are sensible and quantitative. R33 milestones are appropriate, feasible and quantitative. Future studies have been clearly considered. Better evidence could however have been provided to support the idea that indulging cravings is the primary reason for dietary indiscretions in T2DM. The mindfulness intervention to be used was originally designed for binge eating disorder. It is not entirely clear whether the psychological processes relating to eating are similar between binge eating disorder and T2DM. As it is currently written weight loss is an outcome measure but participants can be normal weight. It is unclear whether weight loss would be a goal in these people? The subject exclusion criteria with respect to psychiatric disorders in particular are rather vague. How psychiatric disorders are being assessed is not entirely clear. Blinding could have been better addressed. The Education group might not have equal attention from investigators or home practice. There seem to be no adequate measures of expectancy and subjects might not have their treatment assignments masked. The data analysis plan could have been described in more detail. It was perceived as rather general, somewhat vague and could be more tightly mapped to the proposed aims and hypotheses. In conclusion this is a highly significant and innovative study by a very well qualified group of investigators with access to excellent resources that has the potential to be highly impactful. Enthusiasm was primarily reduced by issues with the study design that in parts was perceived as being rather vague.

DESCRIPTION (provided by applicant): Type 2 diabetes mellitus (T2DM) is the most expensive chronic disease in the U.S. Lifestyle modification is central to T2DM management, but long-term adherence to dietary recommendations is difficult. A key challenge is the difficulty of coping with

cravings for high carbohydrate or sugar-laden foods in an environment where these foods are tempting and widely available. One mechanism by which mindfulness may increase long-term dietary adherence is by better equipping individuals with skills to experience food cravings and difficult emotions without eating in response. Such approaches seek to strengthen abilities to be non- judgmentally aware of, tolerate, and respond skillfully to food cravings and difficult emotions without reacting impulsively or maladaptively. We hypothesize that improved ability to manage food cravings and emotional eating is a key mechanism through which mindfulness-enhancements can improve dietary adherence. We will test mindfulness-based intervention (MBI) components for improving dietary adherence based on our group's recent NCCIH-funded trial testing MBI components for obesity, which showed evidence of improved fasting glucose, lipids, and potentially weight. Although the particular diet employed is not the focus of this study, we plan to use a diet with about 10% of calories from carbohydrate as: (1) it induces a low level of ketone production, which we will use as a biomarker for dietary adherence; (2) prior studies suggest it improves metabolic parameters in T2DM, including glycemic control. METHODS: We will use ecological momentary assessment (EMA) methods to measure eating in response to difficult emotions and/or food cravings. In the R61 phase, we will ensure this measure is appropriate for further testing and assess the impact of the MBI components on our hypothesized behavioral mechanisms in N=45 persons with T2DM. We plan 3 waves of 15 persons each with 12 weekly sessions. All participants will attend an in-person group course providing education on basic behavioral strategies for diet and physical activity. Participants will be randomized to receive this education alone (Ed) or this same material with added MBI components (Ed+MBI). We will also pilot test two levels of intensity of maintenance phase intervention (monthly group meetings alone or supplemented by individualized attention) to prepare them for R33 testing. We plan an R33 phase trial in which 120 persons with T2DM will be randomized (using a 1:2 ratio) to Ed or Ed+MBI conditions and followed for 12 months, including a 9-month maintenance phase. We will test the robustness of the effect of MBI components on our proposed behavioral mechanisms, and on dietary adherence, as well as preliminary effect sizes? on weight and glycemic control. We will use an innovative adaptive intervention design to optimize maintenance phase intensity, which we believe may be key to augment the MBI effects. SIGNIFICANCE: Our studies promise to extend our understanding of how MBIs can improve dietary adherence and have broader implications for understanding the mechanisms of MBIs for other conditions such as addiction.

PUBLIC HEALTH RELEVANCE
Maintaining a healthy lifestyle is central to managing Type 2 Diabetes (T2DM), but long-term adherence to dietary recommendations is difficult. Building on our earlier trial testing a mindfulness-based intervention for weight loss, we plan to optimize and test a mindfulness-based intervention for management of T2DM, with the aim of improving tools to support people with T2DM in following a healthy lifestyle.

CRITIQUE 1:

Significance: 2
Investigator(s): 1
Innovation: 3
Approach: 4
Environment: 1

Overall Impact:

R61: The R61 will test the “ability to adaptively experience food cravings” as the central mechanism whereby mindfulness skills enhance dietary adherence.

R33: The R33 will attempt to further optimize the mechanism through the use of an adaptive intervention strategy whereby intervention intensity is adjusted based on subject response to treatment.

Summary: This is a very solid application from an experienced team of Investigators with a history of successful investigation working in an outstanding research environment. There is great potential for impact on positively enhancing non-medication treatments e.g. lifestyle interventions for type 2 diabetes, a major worldwide health problem. Further, if mindfulness is effective for enhancing capacity to resist food cravings it may be generalizable to management of addiction which is also characterized by craving and which shares some overlapping neurobiology and susceptibility to stress. There are general weaknesses related to the assessment of psychiatric history and detail related to statistical analysis that need to be addressed.

1. Significance:

Strengths

- Type 2 diabetes mellitus and obesity are extremely important public health problems.
- A large and continually growing body of clinical research supports the use of mindfulness-based treatments for stress-related disorders.
- If the investigators achieve the study aims for the R61 and the R33, the potential of lifestyle modifications, a first-line approach to treatment of diabetes and a critical piece of more intensive diabetes treatment, will be greatly strengthened by the addition of mindfulness based treatment to conventional lifestyle modifications.
- The investigators provide ample justification and rationale for the exploratory and implementation phase of the proposed research.

Weaknesses

- No major weaknesses noted.

2. Investigator(s):

Strengths

- The Multiple PI format with complimentary sets of experience and history of effective collaboration between the two principal investigators is a strength.
- Very qualified team with a history of working with the study population and collaboratively with one another.
- Junior members of the team have more than adequate experience to perform their study roles and the experience of working on the project will further contribute to their scientific development.

Weaknesses

- No major weaknesses noted.

3. Innovation:

Strengths

- Use of a mindfulness-based intervention to target stress generally and food cravings specifically is novel and could have broad applicability considering comorbidity of stress-related medical and psychiatric disorders where attention to nutrition is important such as diabetes, hypertension and cardiovascular disease.

- Use of ecological momentary assessment to measure craving and stress triggered eating is novel and likely to yield more accurate data than conventional retrospective methods.
- Use of ketone monitoring as an adherence and biomarker outcome across treatment interventions is novel.
- Use of an adaptive intervention strategy in the R33 phase is novel.

Weaknesses

- No major weaknesses noted.

4. Approach:
Strengths

- Recruitment and retention plans are adequately described.
- The R61 will test the “ability to adaptively experience food cravings” as the central mechanism whereby mindfulness skills enhance dietary adherence. All subjects will receive diet and exercise education (Ed group) and 2/3 of the subjects will receive Ed plus the mindfulness-based intervention (Ed + MBI). The pairing of subjective measures of craving (ecological momentary assessment) with objective measures of behavior (low calorie diet with ketone assessment; 24h dietary recall) as primary outcomes tested across time and between groups is a robust way to evaluate the proposed mechanism. These outcomes are well-described, sufficiently justified, and positioned to provide interpretable results.
- Additional assessments with delayed-discounting task and self-report questionnaires related to impulsivity, stress, and emotion-related eating further enhance the depth and breadth of information derived from the R61 phase and allow for testing of alternative hypotheses depending on the primary outcome data.
- Go/No-Go criteria are sensible and quantitative. They verify the quality of the mindfulness trainer, reliability and validity of EMA, and initial impact of MBI on craving.

Weaknesses

- The subject exclusion criteria with respect to psychiatric disorders in particular are vague and rather arbitrary (“A substance abuse, mental health, or medical condition that, in the opinion of investigators, will make it difficult for the potential participant to participate in the intervention or that may need immediate changes in medical management that will affect study outcome measures”) insofar as what conditions are excluded aside from a history of bulimia or current bulimia.
- How are psychiatric disorders being assessed? There are no specialty trained mental health clinicians on the study.
- Why do the investigators not plan to assess childhood abuse/trauma and adult trauma exposure? Trauma exposure may substantially impact stress reactivity and food craving.
- Along the lines of the above comments, the investigators should consider the potential impact of medications such as atypical antipsychotics, certain anticonvulsants, and certain antidepressants that may increase appetite. Conversely, there are other medications in addition to stimulants (amphetamine salts & methylphenidate) that curtail appetite such as topirimate.
- It would also be important to consider the safety of subjects on significant doses of Beta Blockers participating in the study considering the potential for masked hypoglycemia.
- The data analysis plan, while hypothesis-driven, could use more detail in terms of correcting for multiple testing in secondary data analyses.

5. Environment:

Strengths

- University of California San Francisco generally, and the Osher Center and Center for Health and Community in particular are collectively an outstanding environment for this type of research.
- Location of the study in a large diverse metropolitan area enhances probability of reaching study recruitment goals.
- A letter of support from project consultant, Dr. Laura Saslow is included.

Weaknesses

- No major weaknesses noted.

Protections for Human Subjects:
Acceptable Risks and/or Adequate Protections

- Study risks and protections are adequate. However, it would be helpful for the study physicians to explicitly describe any additional monitoring for individuals on Beta-Blockers and the possibility of masked hypoglycemia on the low calorie diet.

Data and Safety Monitoring Plan (Applicable for Clinical Trials Only):

- Acceptable
    - No concerns.

Inclusion of Women, Minorities and Children:

- Sex/Gender: Distribution justified scientifically
- Race/Ethnicity: Distribution justified scientifically
- Inclusion/Exclusion of Children under 21: Including ages < 21 justified scientifically

Vertebrate Animals:

Not Applicable (No Vertebrate Animals)

R61 Go/No-Go Criteria:

Acceptable

- Go/No-Go Criteria are in general well-defined. However, it would be helpful to have more detail about specific no-go circumstances beyond reaching the quantifiable benchmarks specified by the Go Criteria.

R33 Milestones:

Acceptable

- No concerns, R33 milestones are feasible, quantitative, and reflect R33 aims.

Biohazards:

Not Applicable (No Biohazards)

Resource Sharing Plans:

Not Applicable (No Relevant Resources)

Budget and Period of Support:

Recommend as Requested

CRITIQUE 2:

Significance: 1
Investigator(s): 1
Innovation: 1
Approach: 1
Environment: 1

Overall Impact:

R61 phase: The R61 phase will evaluate the impact of mindfulness-based intervention (MBI) training in addition to lifestyle education (a 10% CHO diet + physical activity) on eating triggered by a) food cravings and b) difficult emotions as measured by ecological momentary assessment (EMA app). It will also test the impact of two levels of intensity of follow-up: a) monthly group visits vs. b) individualized attention. N=45 patients with stable T2DM. Specifically, determine if 1) there is a high (90%) response rate to EMA pings (Go/No Go criteria, 80% or better); 2) the MBI affects the proposed mechanisms (food cravings); and 3) assess feasibility and acceptability of two types of maintenance phase support to ensure readiness for the R33.

R33 phase: The R33 phase will enroll 120 T2DM patients and randomize them in a 1:2 ratio to education alone vs. education plus MBI, including a 9 month maintenance phase, using the optimized intensity of follow-up from the R61 phase. Outcomes include the EMA assessment tested in the R61, weight, and glycemic control. Specifically, the R33 aims to 1) determine whether the proposed mechanisms predict dietary adherence; 2) the MBI intervention improves dietary adherence; and 3) compare the maintenance interventions to optimize maintenance phase approaches in a future trial.

Summary: This is a well thought out project by an established team of investigators using an innovative approach for an important public health problem.

1. Significance:

Strengths

- Type 2 DM is a chronic health condition of considerable public health importance, and its prevalence is rising.
- There is a strong body of research supporting the rationale for this project, including this team’s previous work on a similar intervention in a similar population (obese subjects).
- The proposed project is likely to yield clear answers needed to advance to the next stage of research in this field.

Weaknesses

- No major weaknesses noted.

2. Investigator(s):

Strengths

- The investigators are well suited to conduct this project. They have demonstrated an ongoing record of accomplishments that advance their fields. The leadership approach, governance, and organizational structure are appropriate for this project.
- The investigators have experience and expertise with the intervention, study population, and research methods. They publish in high impact journals. They have experience with successfully recruiting in similar populations.

Weaknesses

- No major weaknesses noted.

3. Innovation:

Strengths

- Clearly identified conceptual model with testable hypotheses.
- Use of EMA, devised by a member of the project team and tested in a prior study to assess eating in response to food cravings and stress.
- Use of blood ketones to measure dietary adherence.
- Use of an adaptive intervention design to test the different maintenance phase approaches.

Weaknesses

- One study has tested MBI for T2DM patients and found similar outcomes to a standard Diabetes Self-Management Education intervention in terms of weight over 3 months. That study had a small sample size, a relatively short follow-up, and did not examine behavioral mechanisms, adherence, or evaluate long-term maintenance approaches.

4. Approach:
Strengths

- The overall strategy, methods, and analyses are well-reasoned and appropriate to accomplish the specific aims of the project. The investigators have considered alternative strategies and potential problems.
- The underlying hypothesized mechanism of action from the R61 phase is appropriate, relevant and rigorously tested. The need for this phase is well justified.
- The investigators have clearly considered the next study following the R33 phase, and considered different options depending on the outcome of the R33 phase (testing weight and HgA1c as outcomes

Weaknesses

- No major weaknesses noted.

5. Environment:

Strengths

- UCSF is an exemplary research environment. The Osher Center is one of the top programs providing and evaluating mindfulness interventions.
- There is reasonable assurance that the target sample size can be enrolled in the proposed time frame. There is an adequate subject pool from San Francisco General Hospital, UCSF University Clinics, and community outreach. Dr. Hecht has experience recruiting large sample sizes for clinical studies.

Weaknesses

- No major weaknesses noted.

Protections for Human Subjects:
Acceptable Risks and/or Adequate Protections

- The diet and mindfulness interventions and monitoring program are low risk.

Data and Safety Monitoring Plan (Applicable for Clinical Trials Only):

- Acceptable
    - There will be a 3 person DSMB.

Inclusion of Women, Minorities and Children:

- Sex/Gender: Distribution justified scientifically
- Race/Ethnicity: Distribution justified scientifically
- Inclusion/Exclusion of Children under 21: Including ages < 21 justified scientifically
- The proposed sample populations are consistent with those of UCSF and are scientifically credible.

Vertebrate Animals:
Not Applicable (No Vertebrate Animals)

R61 Go/No-Go Criteria:
Outstanding

- 1. Identify a teacher with participant ratings of 3.6 or high on a 4 point scale.
- 2. EMA response rate of 80% or higher to ensure this is a feasible way to address eating related to cravings or difficult emotions.
- 3. Assessment that the study protocol is ready for the R33.
- 4. Evidence that the hypothesized mechanism is impacted by the mindfulness intervention as shown by a significant decrease in craving-related eating from baseline to 12-week follow-up. If not, there must be significant improvement on at least 2/5 other measures in the MBI compared with no MBI group to proceed to the R33.

R33 Milestones:
Outstanding

- Timelines and milestones for both phases are clearly described in detail.
- Plans for sample size and timely recruitment are feasible.

Biohazards:

Not Applicable (No Biohazards)

Resource Sharing Plans:

Acceptable

- The last six months of the R33 period will be used in part to prepare a deidentified data set for data sharing.

Budget and Period of Support:

Recommend as Requested

CRITIQUE 3:

Significance: 4
Investigator(s): 3
Innovation: 5
Approach: 5
Environment: 4

Overall Impact:

R61 phase: The team addresses an important public health problem. The investigators show a strong history of working together. They are productive and experts in the field. The environment is appropriate for the type and scope of study. There are several innovative aspects to the project. However, there are a few concerns that may limit the impact on scientific knowledge. Concerns about the theoretical framework of the hypotheses and the choice of the study population and sample size calculations dampen enthusiasm. The rationale for the key measure of mechanism (in the analytic plan and the Go/No-Go criteria) is weak and may have limited relevance for this particular patient group.

R33 phase: As above; a few concerns about the approach limit enthusiasm for the project, including concerns about feasibility of dietary data collection for participants, blinding, randomization, and theoretical framework.

1. Significance:

Strengths

- The team addresses an important public health problem with considerable associated health care costs (Type 2 diabetes).
- If successful, the project could provide important information about a supplemental treatment for diabetes (and obesity).

Weaknesses

- The application notes the “critical importance of developing sustainable approaches to lifestyle intervention in T2DM” citing a study showing ‘faded’ results from a 1 year trial at follow-up

several years later; however, the proposed project collects data only 1 year out from the start of treatment; it would be better if a longer-term outcome is planned.

2. Investigator(s):

Strengths

- Dr. Hecht is the Director of Research, University of California San Francisco Osher Center for Integrative Medicine and has considerable CAM clinical research experience, including a pilot study of mindfulness approaches with type 2 diabetes patients.
- Dr. Peel has extensive experience in research on interactions between psychological stress, eating, metabolism, fat distribution, and cellular aging, although not necessarily with patients with type 2 diabetes.
- The investigative team has a track record of working together and successfully completing projects and publishing results.
- The team contains an endocrinologist with expertise with T2DM.

Weaknesses

- No major concerns noted.

3. Innovation:

Strengths

- The adaptive intervention design is innovative and a great strength.
- The EMA data collection procedure is innovative.
- Use of a carbohydrate restricted diet is a strength, given that this strategy has been gaining support in the diabetes and obesity research community.

Weaknesses

- Using mindfulness-based interventions in diabetes is not innovative.
- Using mindfulness-based interventions to regulate cravings and/or stress reduction is not innovative.

4. Approach:

Strengths

- No major strengths noted.

Weaknesses

- The key mechanistic variable central to the project is participants' "ability to adaptively experience food cravings" (not indulge in them). This assumes that cravings are the primary source of dietary indiscretions in T2DM, that people can recognize when they are having a craving, and that people can accurately report when they ate food because of a craving (the EMA phone tool asks participants, "Did you eat or drink anything in response to a craving today?"). Evidence is not provided to support the idea that indulging cravings is the primary reason for dietary indiscretions in T2DM; in fact, the cited qualitative study lists 12 types of problems, only one of which is 'resisting temptation.' Also, ability to identify cravings may vary widely, and participants may define 'craving' differently than the investigators.
- The mindfulness intervention may not be a good match for the population; the application states that it is based on Dr. Jean Kristeller's intervention which was originally designed for binge

eating disorder. It is not clear whether the psychological processes relating to eating are similar between binge eating disorder and T2DM. Given the concern about the target population above, one wonders if an overweight or disordered eating population would be a better fit for this project.

- There is a concern about the rationale for the target population given the main health outcome measurements; the application states that the Carbohydrate Restricted diet is helpful for glucose control and to lose weight. However, the inclusion criteria say nothing concrete about including participants with poorly controlled diabetes (HbA1c>8) or with an overweight or obese status. It would make more sense if the study targeted individuals with poorly controlled diabetes, for whom a change in HbA1c would be a perfect outcome measure. Alternatively, the investigators could target people with T2DM who are overweight. As it is currently written, weight loss is an outcome measure, but participants can be normal weight; is weight loss a goal in these people?
- The additional materials provided do not clearly describe how the participants report dietary intake data. In the SHINE manual provided, the instructor asks participants to count calories, but does not talk about carbohydrates. The instructions are that participants should pull out calorie counter booklets and calculate calories for each food item; are participants also going to have to report kcal from carbohydrates (the main outcome variable)? This seems difficult for participants.
- Blinding is not adequately addressed; study staff collecting data should be blinded to treatment allocation and participants should be blinded to study team hypothesis (please see https://nccih.nih.gov/research/blog/effective-blinding-plan)
- The power calculation for the R61 is not entirely clear. How did the team decide that a reduction from 50% craving-eating days to 44% craving-eating days is clinically meaningful? Why is the unit days instead of craving-eating incidents? A one-sided test doesn’t seem appropriate, since it is possible that the MBI has an opposite effect than expected (e.g., increasing body awareness ends up increasing awareness of craving, and thus more craving-eating).
- If participants are randomized by individual, and randomization is done in waves, then both interventions must be happening at the same time (needs clarification). Will the Ed portion of the class be mixed participants? if so, the team needs to address possible bleed (participants sharing the MBI across intervention arms).

5. Environment:

Strengths

- Evidence of prior collaboration is provided. Sites have a scientific environment with previous project completion success.
- Adequate laboratory equipment and other physical resources are available.

Weaknesses

- The team included screen shots for the EMA system for collecting data on craving-eating. These appear to depend on Smartphone capability; for example, in one part, participants move a virtual knob on a continuous measure between “not at all stressed” and “extremely stressed.” There is no evidence provided about the percentage of people in the target population who have smartphones, and how this (in essence) inclusion criteria will affect enrollment feasibility.

Protections for Human Subjects:

Acceptable Risks and/or Adequate Protections

- It is a strength that the team is addressing the possibility of hypoglycemia from patients' regular medication regimen of sulfonylureas or insulin. Will study physicians meet with each patient taking diabetes medications before the weekly class, for the entire 12 weeks? This seems important, to monitor medication changes (and home blood glucose measures).
- Participants are instructed to speak to the instructor if they experience dizziness, constipation, diarrhea, weakness, muscle cramps, and that they will be told that their participation is voluntary and that they can withdraw from the study. The team should clarify that participants will have access to an MD to discuss these medical symptoms.

Data and Safety Monitoring Plan (Applicable for Clinical Trials Only):

- Acceptable
    - No concerns other than above.

Inclusion of Women, Minorities and Children:

- Sex/Gender: Distribution justified scientifically
- Race/Ethnicity: Distribution justified scientifically
- Inclusion/Exclusion of Children under 21: Including ages < 21 justified scientifically

Vertebrate Animals:

Not Applicable (No Vertebrate Animals)

R61 Go/No-Go Criteria:

Unacceptable

- Needs clarification. Not all Go/No-Go criteria are quantifiable. The mechanistic Go/No-Go criterion (number 4) is not specific about how this is calculated, what is meant exactly by 'significant,' etc. What are these other 5 measures and how would they be quantified?

R33 Milestones:

Acceptable

Biohazards:

Unacceptable

- Appears to be missing (?)

Budget and Period of Support:

Recommend as Requested

CRITIQUE 4:

Significance: 2
Investigator(s): 1
Innovation: 1
Approach: 3

Environment: 1

Overall Impact:

R61 phase: The applicants propose conducting a R61 trial to compare the effects of Mindfulness + Education (n=30) versus Education only (n=15) in the Type 2 diabetic population. The intervention period is 12 weeks and there are assessments at 0, 3 and 6 months. The goal of the R61 phase is to extend this successfully used intervention in obese but not type 2 diabetic patients. Significant enhancements to the prior studies include use of Ecological Momentary Assessment, the use of a calorie restricted diet for all ED and MBI + ED subjects thereby allowing for an objective measure of blood ketone monitoring to assess compliance with the restricted diet and the innovative use of an adaptive intervention in the MBI+ED group to optimize the maintenance strategy. Overall this portion of the application is carefully prepared and generates a lot of enthusiasm. There are minor issues: The Education group does not appear to have equal attention from investigators or home practice, there seem to be no measures of expectancy, and individual subjects will not have their treatment assignment masked. The Education group may feel less compelled to comply with the study or complete the study if they are aware of the other group receiving more training. The principal investigator and the team have the requisite knowledge to conduct the proposed study, and there is sufficient confidence that the proposed study would provide results necessary to move to the R33 phase and that the Go-No Go criteria are clearly described.

R33 phase: The applicants propose a randomized trial to compare the effects of Mindfulness + Education (n=80) versus Education only (n=40) in a Type 2 diabetic population. Results of the R61 will provide preliminary data on the mechanism related measures and maintenance strategies for use in the R33. The follow-up period in the R33 will be 1 year. There is a possible issue with the Education group failing to complete the study with the long-follow-up period. Expectancy and blinding/masking are minor issues.

Summary: This is a very innovative project from a strong investigative team at a stellar university setting. Given only minor issues, there is great enthusiasm for the applicaiton as written.

CRITIQUE 5:

Overall Impact:

The study design seems reasonable. The analysis plan is very general and vague and does not clearly map to aims and hypotheses. There are many measures being collected in this study and a couple of randomization points; the project could be strengthened by more clearly mapping hypotheses to analysis methods. The Go/No-Go criteria are detailed although criteria for the maintenance phase intervention seem subjective. It was unclear whether mindfulness sessions are held by the same instructor and if group session compositions will always be the same individuals – leading to the potential of a clustering effect that may be unaccounted for in the analysis. Consideration of stratification variables for randomization should be noted for both the R61 and the R33.
For R61 analysis plan:

- One sided tests are not really appropriate for the sake of improving power. Assuming that a t-test was the method for sample size calculation but this was not explicitly stated.
- The study design is not clearly accounted for in the analysis plan. It is unclear how the EMA nature of the data is accounted for in the analysis although it states general use of mixed models for secondary analyses. Sample size calculation is based on percentage of days that responded to cravings – what is the denominator for this calculation? It is stated that it is collected for a 4 day period of once daily EMAs and then 8 times per

  month for the 3-month intervention and then a second 4-day period after the intervention. How is this outcome calculated from this data? How will missing responses be handled?
  - No citations for any analysis methods.
- For R33 analysis plan :
  - The study design is not well accounted for in the analysis plan, there is mention of a SMART trial but this is not reflected in the analysis plan.
  - In the Milestone section for the R33 section, it states specifically that primary analysis for diet adherence is the proportion of keystone measures above threshold. Justification needs to be given for this as an outcome and the clinical meaning of this. Ketone data is collected daily for several weeks and then twice monthly. The analysis should reflect this design. The use of t-test at specific time points for the main comparisons is rather inefficient. More thought and detail needs to be given to the analysis.
  - Sample size calculation does not clearly reflect design and does not map very well to primary aims detailed for the R33.

THE FOLLOWING SECTIONS WERE PREPARED BY THE SCIENTIFIC REVIEW OFFICER TO SUMMARIZE THE OUTCOME OF DISCUSSIONS OF THE REVIEW COMMITTEE, OR REVIEWERS' WRITTEN CRITIQUES, ON THE FOLLOWING ISSUES:

PROTECTION OF HUMAN SUBJECTS (Resume): ACCEPTABLE

INCLUSION OF WOMEN PLAN (Resume): ACCEPTABLE

INCLUSION OF MINORITIES PLAN (Resume): ACCEPTABLE

INCLUSION OF CHILDREN PLAN (Resume): ACCEPTABLE

COMMITTEE BUDGET RECOMMENDATIONS: The budget was recommended as requested.

Footnotes for 1 R61 AT009333-01; PI Name: Hecht, Frederick M

NIH has modified its policy regarding the receipt of resubmissions (amended applications). See Guide Notice NOT-OD-14-074 at http://grants.nih.gov/grants/guide/notice-files/NOT-OD-14-074.html. The impact/priority score is calculated after discussion of an application by averaging the overall scores (1-9) given by all voting reviewers on the committee and multiplying by 10. The criterion scores are submitted prior to the meeting by the individual reviewers assigned to an application, and are not discussed specifically at the review meeting or calculated into the overall impact score. Some applications also receive a percentile ranking. For details on the review process, see http://grants.nih.gov/grants/peer_review_process.htm#scoring.

MEETING ROSTER
National Center for Complementary and Integrative Health Special Emphasis Panel

NATIONAL CENTER FOR COMPLEMENTARY & INTEGRATIVE HEALTH
Mind and Body Interventions
ZAT1 SM (41)
04/08/2016

CHAIRPERSON(S)

COGHILL, ROBERT C, PHD
DIRECTOR OF RESEARCH
DEPARTMENT OF ANESTHESIOLOGY
IMAGING RESEARCH CENTER
CINCINNATI CHILDREN'S HOSPITAL
CINCINNATI, OH 45229

MEMBERS

ANTONOPOULOS, DIONYSIOS, PHD
ASSISTANT PROFESSOR
INSTITUTE FOR GENOMICS
AND SYSTEMS BIOLOGY
DEPARTMENT OF MEDICINE
UNIVERSITY OF CHICAGO
ARGONNE, IL 60439

APKARIAN, APKAR VANIA, PHD
PROFESSOR
DEPARTMENT OF PHYSIOLOGY
FEINBERG SCHOOL OF MEDICINE
NORTHWESTERN UNIVERSITY MEDICAL SCHOOL
CHICAGO, IL 60611

BEATTIE, PAUL F, PHD
CLINICAL PROFESSOR
PROGRAM IN PHYSICAL THERAPY
DEPARTMENT OF EXERCISE SCIENCE
UNIVERSITY OF SOUTH CAROLINA
COLUMBIA, SC 29206

BISHOP, MARK D, PHD
ASSOCIATE PROFESSOR
DEPARTMENT OF PHYSICAL THERAPY
UNIVERSITY OF FLORIDA
GAINESVILLE, FL 32610

BYRNE, DANIEL W, MS
DIRECTOR
QUALITY IMPROVEMENT
AND PROGRAM EVALUATION
DEPARTMENT OF BIOSTATISTICS
VANDERBILT UNIVERSITY SCHOOL OF MEDICINE
NASHVILLE, TN 37232

COFFMAN, CYNTHIA J, PHD
ASSOCIATE PROFESSOR
DEPARTMENT OF BIOSTATISTICS AND BIOINFORMATICS
DUKE UNIVERSITY MEDICAL CENTER
DURHAM, NC 27705

CRISCO, JOSEPH J, PHD
PROFESSOR
THE WARREN ALPERT MEDICAL SCHOOL
BROWN UNIVERSITY
PROVIDENCE, RI 02903

DIMITROVA, ALEXANDRA K, MD
ASSISTANT PROFESSOR
DEPARTMENT OF NEUROLOGY
OREGON HEALTH AND SCIENCE UNIVERSITY
PORTLAND, OR 97239

DUNCAN, LARISSA G, PHD
ASSOCIATE PROFESSOR
HUMAN DEVELOPMENT
AND FAMILY STUDIES
SCHOOL OF HUMAN ECOLOGY
UNIVERSITY OF WISCONSIN, MADISON
MADISON, WI 53706

DUSEK, JEFFERY A, PHD
DIRECTOR OF RESEARCH
INTEGRATIVE HEALTH RESEARCH CENTER
PENNY GEORGE INSTITUTE FOR HEALTH
AND HEALING
ABBOTT NORTHWESTERN HOSPITAL
MINNEAPOLIS, MN 55407

FREWEN, PAUL ANDREW, PHD
ASSOCIATE PROFESSOR
DEPARTMENTS OF PSYCHIATRY
AND PSYCHOLOGY
UNIVERSITY OF WESTERN ONTARIO
LONDON, ON N6A 5A5
CANADA

GABBAY, VILMA, MD
ASSOCIATE PROFESSOR
CHIEF, PEDIATRIC MOOD AND ANXIETY DISORDERS
MOUNT SINAI SCHOOL OF MEDICINE
ASSOCIATE PROFESSOR
NATHAN KLINE INSTITUTE FOR PSYCHIATRIC RESEARCH
NEW YORK, NY 10029

GAYLORD, SUSAN, PHD
PROFESSOR
DIRECTOR, PROGRAM ON INTEGRATIVE MEDICINE
DEPARTMENT OF PHYSICAL MEDICINE
AND REHABILITATION
UNIVERSITY OF NORTH CAROLINA AT CHAPEL HILL
CHAPEL HILL, NC 27599

GEISSER, MICHAEL E, PHD
PROFESSOR
DEPT OF PHYSICAL MEDICINE
AND REHABILITATION
UNIVERSITY OF MICHIGAN
ANN ARBOR, MI 48108

GILLESPIE, CHARLES FREDERICK, MD, PHD
ASSISTANT PROFESSOR
DEPARTMENT OF PSYCHIATRY
AND BEHAVIOURAL SCIENCES
EMORY UNIVERSITY SCHOOL OF MEDICINE
ATLANTA, GA 30322

HOGE, ELIZABETH A, MD
ASSOCIATE PROFESSSOR
SCHOOL OF MEDICINE
GEORGETOWN UNIVERSITY
WASHINGTON, DC 20057

KEMPER, KATHI J, MPH, MD
PROFESSOR AND DIRECTOR
CENTER FOR INTEGRATIVE HEALTH AND WELLNESS
THE OHIO STATE UNIVERSITY
COLUMBUS, OH 43210

KHALSA, SAT BIR SINGH, PHD
ASSISTANT PROFESSOR OF MEDICINE
DIVISON OF SLEEP
AND CIRCOFIAN DISORDERS
BRIGHAM AND WOMEN'S HOSPITAL
BOSTON, MA 02115

KONG, JIANG-TI, MD
INSTRUCTOR
DEPARMENT OF ANESTHESIOLOGY,
PERIOPERATIVE AND PAIN MADICINE
SCHOOL OF MEDICINE
STANDFORD UNIVERSITY
REDWOOD CITY, CA 94063

LIBERZON, ISRAEL, MD
PROFESSOR
DEPARTMENT OF PSYCHIATRY
UNIVERSITY OF MICHIGAN HEALTH SYSTEM
ANN ARBOR, MI 48105

LONGHURST, JOHN C, MD, PHD
PROFESSOR
DEPARTMENT OF MEDICINE
UNIVERSITY OF CALIFORNIA, IRVINE
IRVINE, CA 92697

MARSLAND, ANNA L, RN, PHD
ASSOCIATE PROFESSOR
DEPARTMENT OF PSYCHOLOGY
UNIVERSITY OF PITTSBURGH
PITTSBURGH, PA 15260

MEHLING, WOLF E, MD
ASSOCIATE PROFESSOR
DEPARTMENT OF FAMILY COMMUNITY MEDICINE
OSHER CENTER FOR INTEGRATIVE MEDICINE
UNIVERSITY OF CALIFORNIA, SAN FRANCISCO
SAN FRANCISCO, CA 94115

MELETH, SREELATHA, PHD
ASSOCIATE PROFESSOR
KOGER CENTER
RTI INTERNATIONAL
ATLANTA, GA 30341

MICHENER, LORI ANN, MED, PHD
PROFESSOR
DIVISION OF BIOKINESIOLOGY
AND PHYSICAL THERAPY
UNIVERSITY OF SOUTHERN CALIFORNIA
LOS ANGELES, CA 90089

MONTGOMERY, GUY H, PHD
ASSOCIATE PROFESSOR
DIRECTOR
DEPARTMENT OF ONCOLOGICAL SCIENCES
INTEGRATIVE BEHAVIORAL MEDICINE PROGRAM
MOUNT SINAI SCHOOL OF MEDICINE
NEW YORK, NY 10029

OWEISS, KARIM G, PHD
PROFESSOR
DEPARTMENT OF ELECTRICAL AND COMPUTER
ENGINEERING
HERBERT WESTHEIM COLLEGE OF ENGINEERING
THE MCKNIGHT BRAIN INSTITUTE
UNIVERSITY OF FLORIDA
GAINESVILLE, FL 32611

PRESSMAN, ALICE ROGOT, PHD
INVESTIGATOR
RESEARCH, DEVELOPMENT AND DISSEMINATION
SUTTER HEALTH
WALNUT CREEK, CA 94596

REED, WILLIAM RAY, DC, PHD
ASSOCIATE PROFESSOR
PALMER COLLEGE OF CHIROPRACTIC
PALMER CENTER FOR CHIROPRACTIC RESEARCH
DAVENPORT, IA 52803

REEVES, NORMAN PETER, PHD
ASSISTANT PROFESSOR
COLLEGE OF OSTEOPATHIC MEDICINE
MICHIGAN STATE UNIVERSITY
LANSING, MI 48910

ROSEN, ALLYSON C, PHD
PSYCHOLOGIST
VETERANS AFFAIRS PALO ALTO HEALTH CARE SYSTEM
PALO ALTO, CA 94304

SARON, CLIFFORD DARRYL, PHD
RESEARCH SCIENTIST
CENTER FOR MIND AND BRAIN
UNIVERSITY OF CALIFORNIA, DAVIS
DAVIS, CA 95618

SIEGLE, GREG J, PHD
ASSOCIATE PROFESSOR
DEPARTMENT OF PSYCHIATRY
SCHOOL OF MEDICINE
UNIVERSITY OF PITTSBURGH
PITTSBURGH, PA 15213

SIMMONS, ALAN N, PHD
ASSOCIATE ADJUNCT PROFESSOR
DEPARTMENT OF PSYCHIATRY
DIRECTOR OF RESEARCH FMRI CORE
VETERANS AFFAIRS SAND DIEGO HEALTH CARE SYSTEMS
UNIVERSITY OF CALIFORNIA, SAN DIEGO
SAN DIEGO, CA 92161

TAKANO, TAKAHIRO, PHD
ASSISTANT PROFESSOR
SCHOOL OF MEDICINE AND DENTISTRY
UNIVERSITY OF ROCHESTER MEDICAL CENTER
ROCHESTER, NY 14642

TANG, YI-YUAN, PHD
PROFESSOR
CHAIR IN NEUROSICENCE
DEPARTMENT OF PSYCHOLOGICAL SCIENCES
TEXAS TECH UNIVERSITY
LUBBOCK, TX 79409

TILLISCH, KIRSTEN, MD
ASSOCIATE PROFESSOR
CHIEF, INTEGRATIVE MEDICINE
DIVISION OF DIGESTIVE DISEASES
DAVID GEFFEN SCHOOL OF MEDICINE
UNIVERSITY OF CALIFORNIA, LOS ANGELES
LOS ANGELES, CA 90095

VERNON, HOWARD TERRY, DC, PHD
PROFESSOR
CANADIAN MEMORIAL CHIROPRACTIC COLLEGE
TORONTO, ON M2H 3J1
CANADA

VOGT, BRENT ALAN, PHD
PROFESSOR
DEPARTMENT OF ANATOMY AND NEUROBIOLOGY
BOSTON UNIVERSITY SCHOOL OF MEDICINE
BOSTON, MA 02118

WANG, SHU-MING, MD
ASSOCIATE PROFESSOR
DEPARTMENT OF ANESTHESIOLOGY
YALE UNIVERSITY
NEW HAVEN, CT 06520

WASHBURN, DAVID A, PHD
PROFESSOR
DEPARTMENT OF PSYCHOLOGY
AND NEUROSCIENCE
GEORGIA STATE UNIVERSITY
ATLANTA, GA 30303

WILLIAMS, RHONDA, PHD
ATTENDING PSYCHOLOGIST
REHABILITATION CARE SERVICES
VETERANS AFFAIRS PUGET SOUND HEALTH CARE SYSTEM
SEATTLE, WA 98108

ZHANG, RUIXIN, MD, PHD
ASSISTANT PROFESSOR
DEPARTMENT OF FAMILY AND COMMUNITY MEDICINE
CENTER FOR INTEGRATIVE MEDICINE
UNIVERSITY OF MARYLAND, BALTIMORE
BALTIMORE, MD 21201

SCIENTIFIC REVIEW OFFICER

SCHMIDT, MARTINA, PHD
SCIENTIFIC REVIEW OFFICER
OFFICE OF SCIENTIFIC REVIEW
NATIONAL CENTER FOR COMPLEMENTARY
AND INTEGRATIVE HEALTH
NATIONAL INSTITUTES OF HEALTH
BETHESDA, MD 20892

EXTRAMURAL SUPPORT ASSISTANT

BARTRA, ALFONSO EMMANUEL, BS
EXTRAMURAL SUPPORT ASSISTANT
OFFICE OF SCIENTIFIC REVIEW
NATIONAL CENTER FOR COMPLEMENTARY
AND INTEGRATIVE HEALTH
NATIONAL INSTITUTES OF HEALTH
BETHESDA, MD 20892

PHAM, NANCY P
EXTRAMURAL SUPPORT ASSISTANT
OFFICE OF SCIENTIFIC REVIEW
NATIONAL CENTER FOR COMPLEMENTARY
AND INTEGRATIVE HEALTH
NATIONAL INSTITUTES OF HEALTH
BETHESDA, MD 20892

Consultants are required to absent themselves from the room during the review of any application if their presence would constitute or appear to constitute a conflict of interest.
